# Effect of pharmacological interventions for the treatment of people with post‐COVID‐19 condition: A rapid review

**DOI:** 10.1002/cesm.12001

**Published:** 2023-03-20

**Authors:** K. M. Saif‐Ur‐Rahman, Kavita Kothari, Corinna Sadlier, Frank Moriarty, Ani Movsisyan, Sean Whelan, Petek E. Taneri, Matthew Blair, Gordon Guyatt, Declan Devane

**Affiliations:** ^1^ Evidence Synthesis Ireland and Cochrane Ireland University of Galway Galway Ireland; ^2^ School of Nursing and Midwifery University of Galway Galway Ireland; ^3^ Consultant to Library & Digital Information Networks World Health Organization Kobe Japan; ^4^ Department of Infectious Diseases Cork University Hospital Cork Ireland; ^5^ Department of Medicine University College Cork Cork Ireland; ^6^ School of Pharmacy and Biomolecular Sciences Royal College of Surgeons in Ireland Dublin Ireland; ^7^ Consultant to Methods and Standards Team World Health Organization Geneva Switzerland; ^8^ Department of Clinical Microbiology Galway University Hospital Galway Ireland; ^9^ HRB‐Trials Methodology Research Network University of Galway Galway Ireland; ^10^ Department of Health Research Methods, Evidence and Impact McMaster University Ontario Canada

**Keywords:** PCC, pharmacological interventions, post‐COVID‐19 condition

## Abstract

**Objective:**

Little is known about the treatment of post‐coronavirus disease 2019 (COVID‐19) condition (PCC). This article examines the effectiveness of pharmacological interventions for treating people with PCC.

**Methods:**

We searched Medline, EMBASE, ClinicalTrials. gov, and the International Clinical Trials Registry Platform. Two independent review authors screened citations, extracted data, and assessed the quality of the included studies. Due to heterogeneity in participants, interventions, and outcomes, we synthesized data narratively. We assessed the certainty of evidence using GRADE (Grading of Recommendations, Assessment, Development, and Evaluation).

**Participants:**

People with PCC.

**Interventions:**

Pharmacological interventions include corticosteroids, ivabradine, and inhaled hydrogen.

**Outcome Measures:**

Olfactory function, sinus tachycardia, respiratory function.

**Results:**

We identified 5 completed studies and 41 ongoing studies. Oral corticosteroids and olfactory training had higher olfactory scores after 10 weeks (MD: 5.60, 95% confidence interval [CI]: 1.41 to 9.79). Patients allocated oral corticosteroid, and nasal irrigation demonstrated improved recovery of olfactory function compared with the control group at 40 days (median 60, interquartile range [IQR]: 40 vs. median 30, IQR: 25, *p* = 0.024). Patients allocated to topical corticosteroid nasal spray and olfactory training had improved recovery of olfactory function after 2 weeks (median 7, IQR: 5−10 vs. median 5, IQR: 2−8, *p* = 0.08). Participants allocated to ivabradine had a greater mean reduction in heart rate compared with participants randomized to carvedilol (MD: −4.24, 95% CI: −10.09 to 1.61). Participants allocated to inhaled hydrogen therapy had an improved vital capacity (MD: 0.20, 95% CI: 0.07 to 0.33), forced expiratory volume (MD: 0.19, 95% CI: 0.04 to 0.34), 6‐minute walk test (MD: 55.0, 95% CI: 36.04 to 73.96).

**Conclusions:**

The evidence is of low to very low certainty about the effect of all pharmacological interventions investigated for the treatment of people with PCC. There is currently a significant body of research underway that could expand the evidence to inform treatment decisions on pharmacological interventions for PCC.

## Plain language summary

Can pharmacological interventions help in treating the post‐COVID‐19 condition?

Little is known about the role of pharmacological (treatment using medicinal products) interventions in treating the post‐COVID‐19 condition (PCC) and its symptoms. To support the development of World Health Organization (WHO) guidelines, we conducted a rapid review of the effectiveness of pharmacological interventions for treating people with PCC.

What did we do?

We followed standard rapid review methods. Rapid reviews collect and synthesize evidence in a timely manner. Our population was people with PCC. Our intervention was pharmacological interventions, that is, medicinal products that contain a substance or combination of substances that are intended to treat, prevent, or diagnose a disease. Our outcomes were informed by the published PCC core outcome set.

What did we find?

We searched bibliographic databases and clinical trial registries. We identified five completed studies with 253 participants and 41 ongoing studies. Due to differences in participants, interventions, and outcomes, we summarized information narratively.

Main results

Participants allocated to oral/nasal corticosteroids and olfactory (sense of smell) training, or oral corticosteroids and nasal irrigation had better olfactory scores/function compared with olfactory training alone or with no treatment. Participants allocated to the medicine ivabradine had a greater heart rate reduction compared with those allocated to another medicine, carvedilol. Participants allocated to inhaled hydrogen therapy had improved respiratory function compared with those allocated to ambient air.

Authors' conclusions

Currently, we have low to very low certainty of evidence. This means that certain pharmacological interventions may have benefits for treating people with PCC in certain circumstances, but we are uncertain in other cases. There is much ongoing research targeting important clinical outcomes which could provide evidence for future pharmacological interventions for PCC.

## 1 INTRODUCTION

Severe acute respiratory syndrome, coronavirus 2 (SARS‐CoV‐2) emerged in late 2019 and has caused a global pandemic in the years since. Health systems and societies initially implemented lockdowns and social distancing measures to reduce transmission and minimize acute morbidity and mortality [1]. Evidence on the effectiveness of interventions to treat severe coronavirus disease 2019 (COVID‐19) has been generated through numerous trials, including large pragmatic trials, such as the RECOVERY [2] and SOLIDARITY [3] trials. The development and rollout of COVID‐19 vaccines across many countries have substantially improved outcomes further [4]. However, the decline in severe outcomes associated with acute infection and improved survival has increased focus on the long‐term sequelae in the following weeks and months.

Postviral syndromes have been described following infections with many viruses, including other members of the coronavirus family, with symptoms such as fatigue, mood disturbance, joint pain, and disturbed cognition frequently reported [5, 6, 7]. As reports of post‐COVID‐19 condition (PCC) or “Long COVID” emerged, there was wide variation in reported case definitions, symptomatology, and management. This hampered coordinated research and clinical efforts to manage the condition [8, 9]. Consequently, the World Health Organization (WHO) developed a clinical case definition through Delphi consensus methods [10]. This definition includes (i) a history of probable or confirmed SARS‐CoV‐2 infection and (ii) symptoms, usually 3 months from the onset of acute infection that last at least 2 months without an alternative explanation. Common symptoms include fatigue, shortness of breath, and cognitive symptoms, which generally impact functioning. They may be new or persistent from acute infection and fluctuate over time.

Estimating the prevalence of PCC has proved challenging, as many studies are potentially confounded by low response rates to surveys, variations in the definitions of PCC and associated symptoms, and timing of onset [11]. With these caveats in mind, a recent meta‐analysis derived an estimated global pooled prevalence of PCC of 0.43 (95% confidence interval [CI], 0.39 to 0.46) in COVID‐19‐positive individuals, with fatigue and memory issues representing the most frequently reported symptoms [12]. The potential for those with PCC to experience substantial morbidity is a significant concern for individual patients and health systems that resource their care. There is increasing evidence of PCC being associated with brain‐related morbidity [13, 14] and evidence that COVID‐19 survivors have increased health problems more than the general population by 2 years regardless of initial disease severity [15].

Various nonpharmacological interventions have been evaluated to treat postviral syndromes, including PCC. A recent systematic review identified only five studies that evaluated nonpharmacological interventions. These included exercise (telerehabilitation, pilates, resistance exercise), neuromodulation, or music and cognitive behavioral therapy, with all except the latter showing benefits for their primary outcomes [16].

Little is known about the role of pharmacological interventions in treating PCC and its symptoms. The WHO established a Guideline Development Group (GDG) to construct a living guideline for the clinical management of COVID‐19. To support the development of the new guideline, we report a rapid review of the effectiveness of pharmacological interventions for treating people with PCC. Ideally this, and other reviews, would be developed and maintained as a living review with short interval updates to allow timely identification of potentially effective treatments for patients suffering from PCC. Living reviews are continually updated to incorporate new evidence as it becomes available. However, due to resource constraints, this is not a living review.

## 2 METHODS

### 2.1 Study design and registration

The methods of this rapid review were informed by the rapid review guidance provided by the Cochrane Rapid Reviews Methods Group [17]. Compared with a full systematic review approach, in this rapid review, we searched two databases, and two trial registration platforms as this review was commissioned to inform WHO interim guideline development and had to be produced within a tight timeline (12 weeks). We restricted the language to English only and did not search for gray literature. Two independent review authors screened citations, extracted data, and assessed the quality of the included studies. The review is reported in line with the Preferred Reporting Items for Systematic Review and Meta‐analysis (PRISMA) checklist [18], and the protocol was registered in PROSPERO (International prospective register of systematic reviews). (CRD42022330873). We acknowledge that the PROSPERO registration does not note it as a rapid review.

To provide timely evidence for stakeholder decisions, we consulted with stakeholders in refining the review questions, eligibility criteria, and outcomes of interest. The stakeholders include the WHO GDG members, public health, infectious disease, and pharmacology experts.

### 2.2 Criteria for considering studies for this review

#### 2.2.1 Research question

What is the effect of pharmacological interventions for the treatment of people with post‐COVID‐19 condition?

#### 2.2.2 Population

Our population of interest was patients diagnosed with PCC. As per WHO, “post‐COVID‐19 condition occurs in individuals with a history of probable or confirmed SARS‐CoV‐2 infection, usually three months from the onset of COVID‐19 with symptoms that last for at least two months and cannot be explained by an alternative diagnosis. Common symptoms include fatigue, shortness of breath, cognitive dysfunction, and other symptoms that generally impact everyday functioning. Symptoms may be new‐onset following initial recovery from an acute COVID‐19 episode or persist from the initial illness. Symptoms may also fluctuate or relapse over time” [10]. No minimal number of symptoms is required for the diagnosis, though symptoms involving different organ systems and clusters have been described. A full list of described symptoms and related definitions included in the WHO‐led Delphi consensus statement [10] can be found in the Supporting Information File.

Studies that included patients with PCC with other patient cohorts were excluded unless participants were included as part of a randomized trial using stratified randomization and reported results of patients with PCC separately.

We recognized that studies might investigate the effects of pharmacological interventions in patients identified/classified to have PCC or “long‐COVID” by the study investigators, where such patients do not strictly meet the WHO definition of PCC provided above (e.g., the condition occurring 2 vs. 3 months from the onset of the initial infection). To ensure comprehensiveness in identifying any potentially relevant evidence on this novel condition and to inform interim guideline development, such studies using a broader definition of PCC were included in this version of the rapid review, and participant characteristics, including the PCC definition used in each study, were detailed clearly. If sufficient evidence is available, studies were planned to be categorized and synthesized separately based on how they defined PCC (i.e., different PCC patient populations).

#### 2.2.3 Intervention

We considered pharmacological interventions for treating people with PCC, consisting of medicinal products that contain a substance or combination of substances that are intended to treat, prevent or diagnose a disease or to restore, correct, or modify physiological functions, including chemical entities and biologicals. Specific interventions that were considered, but are not limited to, are:

1. Antivirals: Remdesivir, molnupiravir, nirmatrelvir‐ritonavir, or others.
2. Anticoagulants.
3. Antiplatelets.
4. Corticosteroids.
5. IL6 receptor blockers: Tocilizumab, barilumab, or others.
6. Monoclonal antibodies: Casirivimab‐imdevimab, sotrovimab, or others.
7. Other immunomodulators (i.e., immunostimulants or immunosuppressants).

We specifically excluded interventions consisting of blood, tissue, or cell products; vaccines; advanced therapy medicinal products based on cell or gene therapy; dietary supplements and foods; herbal supplements; probiotics; complementary and alternative medicines; and traditional Chinese medicine. However, a separate review was undertaken to explore the effectiveness of COVID‐19 vaccines on PCC [19].

#### 2.2.4 Comparison

We considered comparisons that compared

1. A pharmacological treatment with no treatment, usual care, or placebo.
2. One pharmacological treatment (Treatment A) with another (Treatment B) from the above list.

#### 2.2.5 Outcomes

The core outcome set for PCC [20] was used in our review and is given below:

*Physiological/clinical outcomes*

1. Cardiovascular functioning, symptoms, and conditions.
2. Fatigue or Exhaustion.
3. Pain.
4. Nervous system functioning, symptoms, and conditions.
5. Cognitive functioning, symptoms, and conditions.
6. Mental functioning, symptoms, and conditions.
7. Respiratory functioning, symptoms, and conditions.
8. Post‐exertion symptom.

*Life impact outcomes*

9. Physical functioning, symptoms, and conditions.
10. Work/occupational and study changes.

*Survival*

11. Survival.

*Recovery*

12. Recovery.

*Other*

13. Safety: Serious adverse events.

We considered the above outcomes assessed at any time point.

#### 2.2.6 Type of studies

We considered studies per Cochrane Effective Practice and Organisation of Care (EPOC) guidelines [21]. Randomized trials, nonrandomized trials, controlled before‐after studies, and interrupted time‐series studies were included. The decision to include a range of study designs in addition to randomized controlled trials was driven by the need to synthesize currently available knowledge while many trials are currently lacking or are in progress. All ongoing studies, including study protocols and registered trials, were considered for any future review updates.

#### 2.2.7 Type of publications

We considered English language papers, available in full text and published in a peer‐reviewed scientific journal or on preprint servers. Trial registrations with published results were also considered for inclusion.

We excluded the following types of studies/publications:

- Animal and invitro studies.
- Modeling studies.
- Systematic reviews and other evidence reviews (although those relevant were used for backward citation tracking).
- Opinion pieces, editorials, conference abstracts.

Conference abstracts were excluded based on a likely lack of sufficient information on the methods, which could preclude the risk of bias assessment.

### 2.3 Search methods for identification

#### 2.3.1 Electronic database

The review team, which includes an information specialist (K. K.), developed the search strategy. We adapted the search strategy from the NICE Guideline 188 (National Institute for Health and Care Excellence). The NICE Guidelines 188 covers the identification, assessment, and management of the long‐term effects of COVID‐19 or long‐COVID. We searched Medline (OVID), EMBASE (Elsevier), ClinicalTrials. gov, and the International Clinical Trials Registry Platform (ICTRP). We did not use a COVID‐19‐specific database as those are a compilation of general medical databases. We found that searching the general medical databases was the most efficient and accurate method for retrieving the research on this complex and large body of evidence. The search was conducted on May 11, 2022, and the search timeline was between January 2020 and May 2022. The complete search strategy for each database has been provided in the Supporting Information File.

We also searched the citation list of relevant systematic reviews of randomized trials, nonrandomized trials, controlled before‐after studies, published protocols, and interrupted time‐series studies.

### 2.4 Data collection and analysis

#### 2.4.1 Software

We screened citations using Rayyan [22]. Rayyan is an open‐source software platform for screening articles in systematic reviews.

#### 2.4.2 Screening

The screening of citations was conducted in two phases. After deduplication, the titles and abstracts of the citations were screened for eligibility independently by two reviewers using Rayyan [22]. While we did not formally pilot screen, training was provided for all reviewers on both the system and inclusion criteria. Disagreements were resolved through a discussion with a third reviewer. The full text of all papers included, or where unclear as to whether the paper was relevant at the title and abstract stage, were reviewed for eligibility independently by two reviewers. Where necessary, disagreements were resolved through discussion with a third reviewer. In addition, a third pharmacist reviewer was consulted, where there was uncertainty about the eligibility of the intervention.

#### 2.4.3 Data extraction

Two reviewers independently extracted data into a pilot‐tested form. Data were extracted based on publication year and title, author name, type of study, sample size, study population characteristics (age, sex, ethnicity, geographic location, and PCC characteristics/symptoms), type of intervention, intervention characteristics (administration route, frequency, dose, and duration) and outcome, including time point of outcome assessment.

#### 2.4.4 Risk of bias assessment

Two reviewers independently assessed the risk of bias for all included studies. We used the nine standard criteria suggested by the Cochrane EPOC guidelines [23] to assess the risk of bias in all randomized trials, nonrandomized trials, and controlled before‐after studies, that is, random sequence generation, allocation concealment, similar baseline outcome measurements, similar baseline characteristics, and incomplete outcome data, knowledge of the allocated interventions adequately prevented during the study, protection against contamination, selective outcome reporting and other risks of bias.

For interrupted time‐series studies, we had planned to assess intervention independent of other changes, the shape of the intervention effect prespecified, intervention unlikely to affect data collection, knowledge of the allocated interventions adequately prevented during the study, incomplete outcome data adequacy, selective outcome reporting and other risks of bias. Discrepancies in assessments of the two independent reviewers were resolved through discussion with a third reviewer.

#### 2.4.5 Measures of treatment effect

For dichotomous data, we extracted the number of participants with the outcome. We planned to calculate the risk ratio (RR) and the corresponding 95% CI. For continuous data, we extracted the mean and standard deviation (SD). When the same outcome(s) was/were measured on the same instrument or could be transferred to the same scale, we calculated the mean difference (MD) on the original scale. We planned to calculate the standardized mean difference (SMD) and corresponding 95% CI for continuous outcomes for studies using different instruments to measure the same outcome.

#### 2.4.6 Data synthesis

We had planned to conduct a random‐effect model meta‐analysis had we identified enough articles with similar interventions, populations, and outcomes. We identified considerable between‐study clinical heterogeneity across participant populations, measured outcomes, measurement tools, and timings of outcome assessment. Thus, we could not pool the results in a meta‐analysis for any of our a priori outcomes. We, therefore, provide a narrative synthesis informed by the Synthesis Without Meta‐analysis (SWiM) guidelines [24]. If there was enough data, we planned to conduct a meta‐analysis and carry out a subgroup analysis to explore heterogeneity. Potential subgroups we identified were:

1. Age: (i) children (under 18 years) versus adults (ii) adults versus older adults (over 60 years) (iii) pregnant people versus not pregnant.
2. Acute illness disease severity (i.e., not requiring hospital admission, requiring hospital admission, and requiring intensive care unit (as adapted from Townsend et al. [25]).
3. Vaccination status.
4. Different doses or duration of treatment.

We had planned to assess publication bias if there were 10 or more studies in the meta‐analysis using the funnel plot and Egger's test.

In narrative synthesis, we organized papers by comparison and outcome.

At the request of the WHO GDG, and to inform their discussions, we also conducted a post‐hoc meta‐analysis of the studies that evaluated the effects of corticosteroids alone or with other interventions on olfactory function. To do this, we converted median and interquartile ranges reported in the studies to means and standard deviations. Given the heterogeneity of outcome instruments, we used the SMD effect measure and a random effects meta‐analysis model.

#### 2.4.7 Sensitivity analysis

We had planned to perform sensitivity analyses to assess the robustness of our findings and explore the impact of methodological issues on effect sizes. This would have involved restricting the analysis to (a) summary effects by different study designs, that is, randomized trials and other designs, and (b) studies judged to be at low risk of bias.

#### 2.4.8 Summary of findings

We summarized the findings using the GRADE approach (Grading of Recommendations, Assessment, Development, and Evaluation) [26]. We have assessed the certainty of the evidence for each outcome based on the study limitations, inconsistency, indirectness, imprecision, and publication bias. The certainty of the evidence was assessed by two independent review authors. Discrepancies were resolved through discussions between the author team.

#### 2.4.9 Role of the funding source

The funder of the study had no role in study design, data collection, data analysis, data interpretation, or writing of the report.

#### 2.4.10 Patient and public involvement

Patients or the public were not involved in the design, or conduct, or reporting, or dissemination plans of this research due to the short timeline of the rapid review. We acknowledge this as a limitation of the review.

## 3 RESULTS

### 3.1 Results of the search

**Figure 1:**
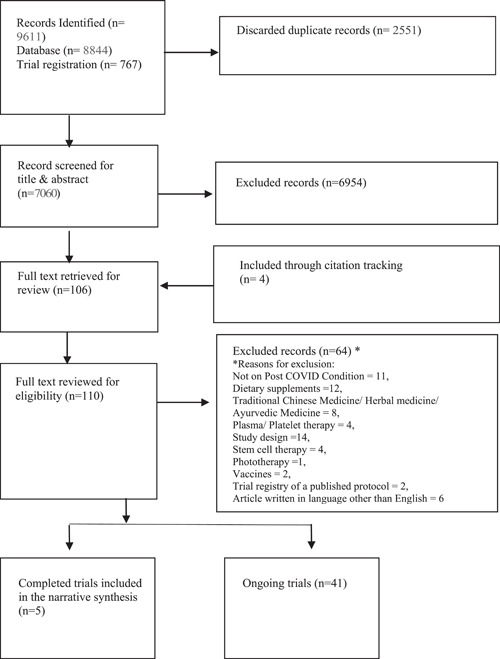
PRISMA flow diagram of the search and screening process. PRISMA, Preferred Reporting Items for Systematic Review and Meta‐analysis.

The PRISMA flow diagram [27], shown in Figure 1, describes the study selection process. We identified 9611 records from the database and the registry searches. After removing 2551 duplicate citations, we screened the title and abstract of the remaining 7060 citations. We excluded 6954 citations at this stage and reviewed the remaining 106 full‐text records. Additionally, we reviewed four full texts that were included through citation tracking. Of the 110 records, we excluded 64 and included 5 published studies. In addition, we identified 41 ongoing studies (1 published protocol and 40 trial registrations). Of the excluded records, 11 were not on PCC, 31 did not evaluate an eligible pharmacological intervention (12 were on dietary supplements, 8 on traditional Chinese medicine, herbal and ayurvedic medicine, 4 on plasma and platelet therapy, 4 on stem cell therapy, 1 on phototherapy, and 2 were on the effect of vaccines), 6 were in Russian, 2 were a trial registry of the published protocol, and 14 were excluded due to study design. The complete list of excluded studies and trial registries is provided in the Supporting Information File.

### 3.2 Included studies

The five studies included in the analysis are described in the following sections.

### 3.3 Characteristics of the included studies

Among the five included studies, one used a controlled before‐after design [28], and four were randomized controlled trials [29, 30, 31, 32]. The studies were conducted in Europe (Belgium, Italy, and the Czech Republic), the Eastern Mediterranean region (Egypt), and South East Asia Region (India). All the studies included men and women aged between 18 and 65 years. The number of participants in the included studies ranged from 18 [29] to 104 [30]. None of the studies included participants with PCC as defined by the WHO [10]. However, as stated in the Section 2, such studies using study investigator definitions of PCC were still considered in this review given the emerging nature of the evidence base. Most of the studies found that the interval between the onset of PCC and COVID‐19 infection and the duration of PCC was unclear. Detailed participant characteristics, including the definition of PCC used in each study, are reported in Table 1.

**Table 1:** Characteristics of included studies (completed).

References	Study design	Population age and gender	Country	Sample size	Outcome reported	Sample characteristics of post‐COVID‐19 condition
Le Bon et al. [28]	Controlled before‐after study	Male and female Mean age 42 (control) Mean age 44 (experimental)	Belgium	Intervention: 9 Control: 18	Olfactory function	“Patients with persistent dysosmia due to COVID‐19. The first olfactory testing session (TDI‐1) was performed in April 2020, 5 weeks after the onset of loss of smell on average.”
Vaira et al. [29]	Randomized controlled trial	Male and female Mean age 42.1 (combined groups)	Italy	Intervention: 9 Control: 9	Olfactory function	“SARS‐CoV‐2 infection recovery from confirmed by at least two negative nasopharyngeal swabs, Connecticut chemosensory clinical research center (CCCRC) test score ≤ 40 (e.g., anosmia or severe hyposmia) at 30 days after clinical onset”
Abdelalim et al. [30]	Randomized controlled trial	Male and female Median age 29	Egypt	Intervention: 54 Control: 54	Olfactory function	“This study was submitted on patients who recently recovered from proven COVID‐19 infection and complaining of anosmia or hyposmia.” Interval between the onset of anosmia and COVID‐19 infection and duration of symptoms is unclear.
Jadhav et al. [31]	Randomized controlled trial	Male and female Mean age 48.8 (combined groups	India	Intervention group 1: 25 Intervention group 2: 25	Sinus tachycardia	“Patients with history recovery from COVID‐19 infection (less than 2 weeks).” Interval between onset of symptoms and COVID‐19 infection and duration of symptoms is unclear.
Botek et al. [32]	Randomized controlled trial	Male and female Mean age Male 39 and Female 37 (control) Mean age Male 45 and Female 41 (experimental)	Czech Republic	Intervention: 26 Control: 24	Respiratory function	“Having a positive RT‐PCR test 21–35 days previously.” Interval between onset of symptoms and COVID‐19 infection and duration of symptoms is unclear.

### 3.4 Description of the intervention and comparison

Three of the included studies examined the effectiveness of corticosteroids for managing post‐COVID olfactory dysfunction [28, 29, 30]. One study compared the effect of carvedilol and ivabradine in controlling post‐COVID sinus tachycardia [31] and one explored the effect of inhaled hydrogen in improving respiratory function [32]. The studies evaluating corticosteroids were heterogeneous regarding the combination of intervention type, route of administration, and dose (Table 2).

**Table 2:** Description of interventions and outcomes of included studies (completed).

References	Intervention	Route of administration, dose, frequency, duration of intervention	Control arm	Timing of outcome assessment	Outcome reported
Le Bon et al. [28]	Oral corticosteroid (methylprednisone) and olfactory training	Oral, 32 mg once daily for 10 days	Olfactory training	10 weeks	Nervous system functioning, symptoms and conditions (change in threshold‐discrimination‐identification (TDI) score) (Mean ± SD)
Vaira et al. [29]	Oral corticosteroid (prednisone) and nasal irrigation with betamethasone (steroid), abroxol (mucolytic) and rinazine (decongestant)	Oral, 1 mg/kg, with a tapering dose for 15 days; Nasal irrigation for 15 days	No treatment	20 days (5 days posttreatment) and 40 days (35 days posttreatment)	Nervous system functioning, symptoms and conditions (change in olfactory score (CCCRC—Connecticut chemosensory clinical research center (CCCRC) test score)) (Median (IQR)
Abdelalim et al. [30]	Topical corticosteroid nasal spray (mometasone furoate) and olfactory training	Nasal spray, once daily, 2 puffs (100 µg in total) for 3 weeks	Olfactory training	3 weeks	Nervous system functioning, symptoms and conditions (visual analog scale [VAS]‐smell score) (Median (IQR)
Jadhav et al. [31]	Group 1: Carvedilol Group 2: Ivabradine	Carvedilol: Oral, twice daily, 3.125–12.5 mg for 5 days Ivabradine: Oral, twice daily, 5–10 mg for 5 days	Comparison between groups	5 days	Cardiovascular functioning, symptoms, and conditions (reduction of heart rate) (Mean difference ± SE)
Botek et al. [32]	Inhaled hydrogen	Inhaled, twice daily for 60 min, 300 mL/min for 14 days	Inhaled ambient air	14 days	Respiratory functioning, symptoms, and conditions (respiratory function) (Mean ± SD)

The controlled before‐after study [28] used oral corticosteroid (methylprednisone) and olfactory training as the intervention. Methylprednisone was administered as a 32 mg oral dose daily for 10 days. The control arm received olfactory training only. Similar to this study, the randomized controlled trial by Vaira et al. [29] evaluated the effects of corticosteroids and olfactory training.However, they evaluated topical corticosteroid nasal spray (mometasone furoate) used once daily (2 puffs, 100 µg in total) for 3 weeks. The control group received olfactory training only. The third study to evaluate corticosteroids [30] evaluated oral corticosteroid (prednisone) and nasal irrigation with betamethasone (steroid), abroxol (mucolytic), and rinazine (decongestant). Oral corticosteroid was prescribed at 1 mg/kg, with a tapering dose for 15 days. Nasal irrigation was continued for 15 days. The control group received no treatment.

The open‐labeled randomized control trial [31] compared the effectiveness of carvedilol prescribed orally, twice daily, 3.125–12.5 mg for 5 days with ivabradine prescribed orally, twice daily, 5–10 mg for 5 days. Botek et al. [32] used a randomized controlled trial to evaluate the effects of inhaled hydrogen twice daily for 60 min, 300 mL/min for 14 days, compared with inhaled ambient air. Table 2 describes the interventions and outcomes of the included studies.

### 3.5 Description of the outcome and measurements

Three studies reported on olfactory function [28, 29, 30], which mapped to our a priori outcome domain of “nervous system functioning, symptoms, and conditions.” We mapped the outcome of sinus tachycardia [31] with our a priori outcome domain “cardiovascular functioning, symptoms, and conditions.” The other study [32] reported respiratory function, which mapped to our a priori outcome domain of “respiratory functioning, symptoms, and conditions.” Despite having the same outcome category, there was heterogeneity in the outcome measures used in the studies reporting olfactory function. The studies used change in threshold‐discrimination‐identification (TDI) score after 10 weeks [28], change in olfactory score measured by CCCRC (Connecticut chemosensory clinical research Center) test score after 5 days and 35 days of treatment [29], and visual analog scale (VAS)‐smell score after 3 weeks [30] to measure the improvement of olfactory function.

Jadhav et al. [31] reported the effect of carvedilol and ivabradine on sinus tachycardia. The timing of the outcome assessment was after 5 days of treatment. Botek et al. [32] measured respiratory function using forced vital capacity, forced expiratory volume, oxygen saturations at rest, change in subjective assessment of dyspnea, change in 6‐minute walk test (minutes), change in oxygen saturation (%) after 6‐minute walk test and change in the rate of perceived exertion as reported outcome measures after 2 weeks of treatment.

Two studies reported the recovery of symptoms [29, 30], and another two reported side effects [31, 32].

No study reported outcome data on pain; cognitive functioning, symptoms, and conditions; mental functioning, symptoms, and conditions; postexertion symptom; life impact outcomes (physical functioning, symptoms, and conditions, work/occupational and study changes), and survival.

### 3.6 Characteristics of the ongoing studies

We identified 41 ongoing studies (1 published protocol and 40 trial registries). None of the trial registry entries included results. The ongoing studies mentioned a variety of pharmacological interventions including various routes (oral, intravenous, inhaled, or topical) and small molecule agents, biologic agents, and medical gases. The most common treatments under investigation included systemic corticosteroids (*n* = 5), nasal corticosteroids (*n* = 4), hyperbaric or concentrated oxygen (*n* = 4), (deu)pirfenidone (*n* = 3), and colchicine (*n* = 3). The outcomes included in the trial registration mapped to our a priori outcomes, including cardiovascular functioning, symptoms and conditions (*n* = 2), fatigue or exhaustion (*n* = 5), nervous system functioning, symptoms and conditions (*n* = 12), cognitive functioning, symptoms and conditions (*n* = 6), mental functioning, symptoms and conditions (*n* = 4), respiratory functioning, symptoms and conditions (*n* = 18), physical functioning, symptoms and conditions (*n* = 4), survival (*n* = 1), and recovery (*n* = 1). Some trial registrations mentioned multiple outcome domains and specific outcomes. Detailed characteristics of the ongoing trials are provided in the Supporting Information File.

### 3.7 Risk of bias of included studies

**Figure 2:**
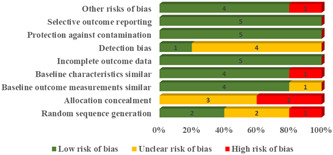
Risk of bias in included studies.

The risk of bias of included studies is summarized in Figure 2. Random sequence generation was assessed as a low risk of bias in two studies [29, 32], as unclear in two studies [30, 31], and as high risk of bias in one study [28]. Allocation concealment was assessed as an unclear risk of bias in three studies [29, 30, 32] and assessed as a high risk of bias in two studies [28, 31]. Baseline outcome measurement was assessed as low risk of bias in four studies [28, 29, 30, 32] and unclear in one study [31]. The similarity of baseline characteristics was assessed as a low risk of bias in four studies [28, 29, 30, 32] and high risk in one study [31], which did not report the baseline measurements separately for the two groups. All the included studies were reported as low risk of bias for incomplete outcome data. Detection bias was assessed as an unclear risk of bias in four studies [28, 30, 31, 32] and low risk of bias in one study [29], which noted that the statistician who analyzed the data was blinded to the patient allocation group. All five included studies were assessed as having a low risk of bias for protection against contamination and selective outcome reporting. The “other bias” domain was assessed as high risk in one study [28] where the participants volunteered to be allocated to the intervention and control group, and low risk of bias for four studies [29, 30, 31, 32]. The risk of bias of individual studies is provided in the Supporting Information File.

### 3.8 Effect of interventions

We identified considerable between‐study clinical heterogeneity across participant populations, measured outcomes, measurement tools, and timings of outcome assessment. Therefore, we provide a narrative summary of five comparisons with their respective summary of findings tables (Tables 3A, 3B, 3C, 3D, 3E). For each study, we recalculated, where possible, the corresponding 95% CI on the original instrument scale for continuous data. Where this was not possible, for example, where data were insufficient to inform these calculations, we presented the data using summary estimates reported in individual studies.

1. Oral corticosteroid (methylprednisone) and olfactory training compared with olfactory training only
  a. *Outcome domain*—*Nervous system functioning, symptoms, and conditions*.
  b. *Outcome*—*Olfactory function*.

One study [28] reported the effects of oral corticosteroid (methylprednisone) and olfactory training compared with olfactory training only on change in TDI score. The oral corticosteroid (methylprednisone) and olfactory training group had higher (improved) olfactory change scores after 10 weeks compared with the olfactory training group after 10 weeks (MD: 5.60, 95% CI: 1.41 to 9.79, 1 study, 27 participants) but the evidence is of very low certainty.

2. Oral corticosteroid (prednisone) and nasal irrigation with betamethasone (steroid), abroxol (mucolytic), and rinazine (decongestant) compared with no treatment
  a. *Outcome domain*—*Nervous system functioning, symptoms, and conditions*.
  b. *Outcome*—*Olfactory function*.

**Table 3A:** Summary of findings—Oral corticosteroid (methylprednisone) and olfactory training compared with olfactory training for people with the post‐COVID‐19 condition.

Outcome	Study results and measurements	Absolute effect estimates		Certainty of the Evidence (Quality of evidence)	Plain language summary
		Olfactory training	Oral corticosteroid (methylprednisone) and olfactory training		
Olfactory function *[Nervous system functioning, symptoms, and condition]*	Measured by: Change in Threshold‐discrimination‐identification (TDI) score (scale: 1 to 48, ≥30.75 (normosmic) −<30.75 (dysosmic), higher better) Minimal important difference: 5.5 Based on data from 27 participants in 1 controlled before‐after study Follow‐up: 10 weeks	2.1 (5.5) Mean (SD)	7.7 (5.1) Mean (SD)	Very low Due to very serious risk of bias and serious imprecision^a^	We are uncertain whether oral corticosteroid (methylprednisone) and olfactory training improves or worsens olfactory function
		Difference: 5.6 higher (MD) (CI 95% 1.41 higher to 9.79 higher)			

Population: Post‐COVID‐19 condition.

Intervention: Oral corticosteroid (methylprednisone) and olfactory training.

Comparator: Olfactory training.

Abbreviations: COVID‐19, coronavirus disease 2019; TDI, threshold‐discrimination‐identification.

a Risk of Bias: very serious. Inadequate sequence generation/generation of comparable groups, resulting in potential for selection bias, inadequate concealment of allocation during randomization process, resulting in potential for selection bias, inadequate/lack of blinding of outcome assessors, resulting in potential for detection bias; Imprecision: serious. Low number of patients, only data from one study; in addition, the 95% CI lower limit crosses the minimally important difference threshold of 5.5 points in TDI score.

**Table 3B:** Summary of findings—Effect of pharmacological interventions for the treatment of people with the post‐COVID‐19 condition.

Outcome	Study results and measurements	Absolute effect estimates			Certainty of the Evidence (Quality of evidence)	Plain language summary
		No treatment		Oral corticosteroid (Prednisone) and nasal irrigation		
Olfactory function *[Nervous system functioning, symptoms, and condition]*	Measured by: Connecticut chemosensory clinical research center (CCCRC) test score (scale: 0‐100, higher better) Based on data from 18 participants in 1 randomized controlled trialFollow‐up: 20 days	10 (15) Median (IQR)		40 (45) Median (IQR)	Very low Due to serious risk of bias and very serious imprecision^a^	We are uncertain whether oral corticosteroid (prednisone) and nasal irrigation with betamethasone (steroid), abroxol (mucolytic) and rinazine (decongestant) improves olfactory function
		Difference: 30 higher (Median) (insufficient data to calculate CI of median difference)				
	Measured by: Connecticut chemosensory clinical research center (CCCRC) test score (scale: >40, higher better) Based on data from 18 participants in 1 randomized controlled trial Follow‐up: 40 days	30 (25) Median (IQR)		60 (40) Median (IQR)		
		Difference: 30 higher (Median)(insufficient data to calculate CI of median difference)				

Population: Post‐COVID‐19 condition.

Intervention: Oral corticosteroid (Prednisone) and nasal irrigation with betamethasone (steroid), abroxol (mucolytic), and rinazine (decongestant).

Comparator: No treatment.

Abbreviation: COVID‐19, coronavirus disease 2019.

^a^ Risk of Bias: serious. Inadequate concealment of allocation during randomization process, resulting in potential for selection bias; Imprecision: very serious. Very low number of patients; only data from one study.

**Table 3C:** Summary of findings—Effect of pharmacological interventions for the treatment of people with post‐COVID‐19 condition.

Outcome	Study results and measurements	Absolute effect estimates			
		Olfactory training	Topical corticosteroid nasal spray (mometasone furoate)	Certainty of the Evidence (Quality of evidence)	Plain language summary
Olfactory function *[Nervous system functioning, symptoms, and condition]*	Measured by: Visual analog scale (VAS)‐smell score (scale: 0–10, higher better) Based on data from 108 participants in 1 randomized controlled trial Follow‐up: 1 week	2 (1−5) Median (IQR)	5 (2−5) Median (IQR)	Low Due to serious risk of bias, and serious imprecision^a^	Topical corticosteroid nasal spray (mometasone furoate) may improve olfactory function
		Difference: 3 higher (Median) (insufficient data to calculate CI of median difference)			
	Follow‐up: 2 weeks	5 (2−8) Median (IQR)	7 (5−10) Median (IQR)		
		Difference: 2 higher (Median) (insufficient data to calculate CI of median difference)			
	Follow‐up: 3 weeks	10 (5−10) Median (IQR)	10 (9−10) Median (IQR)		
		Difference: 0 (Median) (insufficient data to calculate CI of median difference)			

Population: Post‐COVID‐19 condition.

Intervention: Topical corticosteroid nasal spray (mometasone furoate).

Comparator: Olfactory training.

Abbreviation: COVID‐19, coronavirus disease 2019.

^a^ Risk of Bias: serious. Inadequate sequence generation/generation of comparable groups, resulting in potential for selection bias, inadequate concealment of allocation during randomization process, resulting in potential for selection bias, inadequate/lack of blinding of outcome assessors, resulting in potential for detection bias; Imprecision: serious. Low number of patients; only data from one study.

**Table 3D:** Summary of findings—Effect of pharmacological interventions for the treatment of people with the post‐COVID‐19 condition.

Outcome	Study results and measurements	Absolute effect estimates		Certainty of the Evidence (Quality of evidence)	Plain language summary
		Carvedilol	Ivabradine		
Sinus tachycardia [Cardiovascular functioning, symptoms and conditions]	Measured by: Mean Heart Rate (MHR) (lower better) Based on data from 50 participants in 1 randomized controlled trial Follow‐up: 5 days	32.92 (11.65) Mean (SD)	37.16 (9.32) Mean (SD)	Very low Due to very serious risk of bias and serious imprecision^a^	We are uncertain whether ivabradine improves or worsens sinus tachycardia in comparison to Carvedilol
		Difference: 4.24 lower (MD) (CI 95% −10.09 lower to 1.61 higher)			

Population: Post‐COVID‐19 condition.

Intervention: Ivabradine.

Comparator: Carvedilol.

Abbreviation: COVID‐19, coronavirus disease 2019.

^a^ Risk of Bias: very serious. Inadequate sequence generation/generation of comparable groups, resulting in potential for selection bias, Inadequate concealment of allocation during randomization process, resulting in potential for selection bias, Inadequate/lack of blinding of outcome assessors, resulting in potential for detection bias, unclear risk of bias for similar baseline outcome measurements, high risk of bias for similar baseline characteristics; Imprecision: serious. Low number of patients; only data from one study.

**Table 3E:** Summary of findings—Effect of pharmacological interventions for the treatment of people with post‐COVID‐19 condition.

Outcome	Study results and measurements	Absolute effect estimates		Certainty of the Evidence (Quality of evidence)	Plain language summary
		Inhaled ambient air	Inhaled hydrogen therapy		
Respiratory function *[Respiratory functioning, symptoms, and conditions]*	Measured by: FVC—forced vital capacity (L) (higher better) Based on data from 50 participants in 1 randomized controlled trial Follow‐up: 14 days	−0.01 (0.22) Mean (SD)	0.19 (0.24) Mean (SD)	Very low Due to serious risk of bias, serious imprecision and indirectness^a^	We are uncertain whether hydrogen therapy improves or worsens respiratory function
		Difference: 0.20 higher (MD) (CI 95% 0.07 higher to 0.33 higher)			
	Measured by: FEV1—forced expiratory volume in the first second (L) (higher better) Based on data from 50 participants in 1 randomized controlled trial Follow‐up: 14 days	−0.08 (0.27) Mean (SD)	0.11 (0.28) Mean (SD)		
		Difference: 0.19 higher (MD) (CI 95% 0.04 higher to 0.34 higher)			
	Measured by: 6 MWT—6‐min walking test (m) (higher better) Minimal important difference: 30 m Based on data from 50 participants in 1 randomized controlled trial Follow‐up: 14 days	9 (29) Mean (SD)	64 (39) Mean (SD)		
		Difference: 55 higher (MD) (CI 95% 36.04 higher to 73.96 higher)			
	Measured by: Oxygen saturation at rest (%) (higher better) Based on data from 50 participants in 1 randomized controlled trial Follow‐up: 14 days	0.2 (0.7) Mean (SD)	0.2 (0.7) Mean (SD)		
		Difference: 0.00 (MD) (CI 95% −0.39 lower to 0.39 higher)			
	Measured by: Oxygen saturation during 6‐min walking test (%) (higher better) Based on data from 50 participants in 1 randomized controlled trial Follow‐up: 14 days	1.2 (2.7) Mean (SD)	1.5 (2.7) Mean (SD)		
		Difference: 0.30 (MD) (CI 95% −1.20 lower to 1.80 higher)			
Dyspnea *[Respiratory functioning, symptoms, and conditions]*	Measured by: Dyspnea (points): MRC dyspnea scale (Grade 0–4) (lower better) Based on data from 50 participants in 1 randomized controlled trial Follow‐up: 14 days	**−**0.8 (0.5) Mean (SD)	−0.8 (0.8) Mean (SD)	Low Due to serious risk of bias and serious imprecision^b^	Hydrogen therapy may result in little to no difference in dyspnea
		Difference: 0.00 (MD) (CI 95% 0.37 lower to 0.37 higher)			

Population: Post‐COVID‐19 condition.

Intervention: Inhaled hydrogen therapy.

Comparator: Inhaled ambient air.

Abbreviation: COVID‐19, coronavirus disease 2019.

^a^ Risk of Bias: serious. Inadequate concealment of allocation during the randomization process, resulting in potential for selection bias, Inadequate/lack of blinding of outcome assessors, resulting in potential for detection bias; Imprecision: serious. Low number of patients; only data from one study. In addition, the 95% CI lower limit crosses the minimally important difference threshold of 30 m for the 6‐min walking test. Indirectness: downgraded one level for use of surrogate outcomes.

^b^ Risk of Bias: serious. Inadequate concealment of allocation during randomization process, resulting in potential for selection bias, Inadequate/lack of blinding of outcome assessors, resulting in potential for detection bias; Imprecision: serious. Low number of patients; only data from one study.

One study [29] reported a change in the CCCRC test score. The authors reported medians and interquartile ranges (IQR). The study was small (*n* = 18), and therefore instead of assuming normality of data distribution and translating medians and IQRs to means and SDs, we report data originally reported by authors as medians and IQRs. Patients in the intervention group demonstrated improved recovery of olfactory function compared with patients in the control group at 20 days (median 40, IQR 45 vs. median 10, IQR 15, *p* = 0.011, 1 study, 18 participants) and 40 days (median 60, IQR 40 vs. median 30, IQR 25, *p* = 0.024, 1 study, 18 participants) but the evidence is of low certainty.

3. Topical corticosteroid nasal spray (mometasone furoate) and olfactory training compared with olfactory training only.
  a. *Outcome domain*—*Nervous system functioning, symptoms, and conditions*.
  b. *Outcome*—*Olfactory function*.

One study [30] reported the change in VAS smell score using medians and interquartile ranges. This was a small study (*n* = 108), and therefore instead of assuming normality of data distribution and translating medians and IQRs to means and SDs, we report data originally reported by authors as medians and IQRs. There was an improvement in the intervention groups in the recovery of olfactory function after 1 week (median 5, IQR 2−5 vs. median 2, IQR 1−5, *p* = 0.10, 1 study, 108 participants), and 2 weeks (median 7, IQR 5−10 vs. median 5, IQR 2−8, *p* = 0.08, 1 study, 108 participants) but the differences were not statistically significant. There was no difference between the groups after 3 weeks (median 10, IQR 9−10 vs. median 10, IQR 5−10, *p* = 0.16, 1 study, 108 participants). Overall, the evidence is of very low certainty.

4. Effect of carvedilol versus ivabradine.
  a. *Outcome domain*—*Cardiovascular functioning, symptoms, and conditions*.
  b. *Outcome*—*Sinus tachycardia (mean heart rate [MHR])*.

One study [31] reported that participants allocated to ivabradine had a greater mean reduction in heart rate compared with participants randomized to carvedilol, but this difference was not statistically significant (MD: −4.24, 95% CI: −10.09 to 1.61, 1 study, 50 participants) and the evidence is of very low certainty.

5. Effect of inhaled hydrogen compared to inhaled ambient air.
  a. *Outcome domain*—*Respiratory functioning, symptoms, and conditions*.
  b. *Outcome*—*Respiratory function as reported by*: FVC—forced vital capacity (L);
    FEV1—forced expiratory volume in the first second (L);
    Dyspnea (points);
    SpO2rest—oxygen saturation in resting condition (%);

6. MWT—6‐minute walk test (m);
  SpO2walk—oxygen saturation during 6‐min walk test (%);
  RPE—rate of perceived exertion (points).

One study [32] reported that participants allocated to the inhaled hydrogen therapy group had an improved vital capacity (L), (MD: 0.20, 95% CI: 0.07 to 0.33, 1 study, 50 participants), forced expiratory volume (L), (MD: 0.19, 95% CI: 0.04 to 0.34, 1 study, 50 participants), 6‐minute walk test (m), (MD: 55.0, 95% CI: 36.04 to 73.96, 1 study, 50 participants). There was an improvement in oxygen saturation during the 6‐minute walk test (%), (MD: 0.30, 95% CI: −1.2 to 1.8, 1 study, 50 participants) and rate of perceived exertion (points) (MD: 0.20, 95% CI: −1.08 to 1.48, 1 study, 50 participants). However, these changes were not statistically significant. There was no improvement in dyspnea (points), (MD: 0.00, 95% CI: –0.37 to 0.37, 1 study, 50 participants), and oxygen saturation at rest (%), (MD: 0.00, 95% CI: −0.39 to 0.39, 1 study, 50 participants). For all outcomes, the evidence is of low certainty.

At the request of the WHO GDG, and to inform their discussions, we also conducted a meta‐analysis of the three studies that evaluated the effects of corticosteroids alone or with other interventions on olfactory function. To do this, we converted median and interquartile ranges reported in two studies to means and standard deviations using the work of Wan et al. [33] Given the heterogeneity of outcome instruments, we used the SMD effect measure and a random effects meta‐analysis model. To help with interpretation, we re‐expressed the SMD and associated CI in the units of the TDI instrument used by Le Bon et al. [28] following guidance from Schünemann et al. [34]. Given the relatively small sample size of the included studies, and to better reflect among‐person variation in practice, we calculated an absolute difference in means by multiplying the pooled SMD by an estimate of the SD (4.20) taken from normative data based on a large‐scale observational sample (*n* = 9139) [35]. We present this analysis and its associated Summary of Findings in Table 3F in the Supporting Information File. With regard to the target of the certainty of evidence rating [36], we used a minimally contextualized approach and rated certainty in relation to the null effect.

## 4 DISCUSSION

Following a comprehensive search and systematic inclusion process, we identified five completed and published studies and 41 ongoing trials investigating the effects of pharmacological interventions on the treatment of people with PCC. Among the five included studies, three focused on our a priori outcome nervous system functioning, symptoms, and conditions (olfactory function), one reported cardiovascular functioning, symptoms, and conditions (sinus tachycardia), and one reported respiratory functioning, symptoms, and conditions. The evidence is of low to very low certainty about the effect of corticosteroids (oral or tropical, with or without olfactory training) on olfactory function. Compared with carvedilol, ivabradine may have little to no effect on reducing heart rate in patients with post‐COVID sinus tachycardia, but the evidence is of very low certainty. We are also uncertain about the effects of inhaled hydrogen therapy on improving respiratory function.

Relatively few of our a priori outcomes, which reflect the most common symptoms experienced by people suffering from PCC, were reported in included studies. For example, these studies did not report fatigue or exhaustion, pain, cognitive functioning, symptoms and conditions, mental functioning, symptoms and conditions, post‐exertion symptom, physical functioning, symptoms and conditions, work/occupational and study changes, and survival. However, the 41 identified and included ongoing trials seek to report the majority of our a priori outcomes with the exception of pain, post‐exertion symptoms, work/occupational and study changes.

### 4.1 Overall completeness and applicability of evidence

We identified a limited number of completed studies but a relatively large number of ongoing trials exploring the effect of pharmacological interventions on treating people with PCC. Among the included studies, three were conducted in Europe, one in the South East Asia Region, and one in the Eastern Mediterranean Region. This may hinder the applicability of the findings in other regions. None of the studies included participants with PCC as defined by the WHO, however, some may have developed their study protocols or commenced recruitment before this definition was published. Future research focusing on this topic should consider the WHO definition of PCC.

We intended to explore the effects of a range of pharmacological interventions and examine the effects of interventions on PCC using a core outcome set [20]. However, included published studies report a narrow range of interventions addressing a similarly narrow range of clinical symptoms. The ongoing studies cover a broader range of pharmacological interventions and seek to report a broader range of clinically relevant outcomes. Most of the ongoing studies are due to be completed in 2023.

### 4.2 Quality of the evidence

We assessed the risk of bias in included studies using the Cochrane EPOC guidelines for assessing the risk of bias in randomized trials, nonrandomized trials, and controlled before‐after studies. We assessed the certainty of the evidence using the GRADE approach. We could not assess the publication bias as the number of included studies was insufficiently small. The certainty of the evidence was downgraded from low to very low for one outcome (olfactory function—nervous system functioning, symptoms, and conditions) and very low for one outcome (sinus tachycardia—cardiovascular functioning, symptoms, and conditions) and low for one outcome (respiratory function—respiratory functioning, symptoms, and conditions). This was due to the study limitations including a high/unclear risk of bias for random sequence generation, high/unclear risk of bias for allocation concealment, unclear risk of bias for blinding of outcome assessors, unclear risk of bias for similar baseline outcome measurements, high risk of bias for similar baseline characteristics, as well as unexplained heterogeneity (inconsistency) of results and, imprecision (as results were based on a limited number of small studies).

### 4.3 Potential biases in the review process

We searched only two databases and two trial registration platforms. This may limit the inclusion of potentially eligible studies. However, we considered the major databases in this rapid review and conducted extensive citation tracking of the included papers and related systematic reviews. In addition, one of the trial registration platforms (ICTRP) allows searching of 18 national/regional trial registries, improving the comprehensiveness of our search. This increases the likelihood that we have not missed important papers or studies that might change conclusions.

We did not search COVID‐specific databases. To capture the literature on this topic, our complex multi‐line search used two concepts (COVID‐19 and long‐COVID) as well as a broad study design filter. One of the main reasons to not use a COVID‐specific database was the unpredictability of how the study filter would perform when translated to the COVID‐specific database (such as the WHO COVID‐19 Research Database). There were also further difficulties in using this complex multi‐line search on COVID‐specific databases, which does not allow for multi‐line searches nor complex proximity searches. Adjusting the search to the COVID‐specific database would cause losses in sensitivity and specificity. In summary, we found that searching the general medical databases was the most efficient and accurate method for retrieving the research on this complex and large body of evidence even though extra time was taken to build the searches across the medical databases used and deduplicate within them. We do not see any reason why bias could have been introduced using the recommended method of using two general scientific resources. We further avoided bias through thorough searching of the trial registries.

Another limitation is the inclusion of papers written in English only. We have excluded six articles written in Russian. The very low to low certainty of evidence from this review and the presence of a relatively large number of ongoing studies suggest that the conclusion of this review may be changed in the future.

### 4.4 Agreements and disagreements with other studies or reviews

We conducted a comprehensive search and sought to identify any related systematic reviews during the screening process but found none. We identified one scoping review [37] describing the registered clinical trials investigating treatments of “long COVID.” The authors defined “long COVID” as “sequelae of COVID‐19 infection persisting beyond the resolution of the acute phase of disease.” The scoping review identified 59 clinical trial registration records. However, most of the trial registrations were on nonpharmacological interventions. The authors reported 23 trials of rehabilitation/therapy including virtual and telemedicine components and psychotherapy. The scoping review identified nine trials evaluating pharmacotherapies. The authors also considered biological treatments, dietary supplements, homeopathic/alternative medicine regimes, and radiotherapy. Similar to our review, the authors note considerable heterogeneity regarding the definition or presentation of PCC. The authors identified the terms such as “long COVID,” “post‐COVID‐19 syndrome (PCS),” post‐acute sequelae of COVID‐19 (PACS), and “SARS‐CoV‐2 post‐viral fatigue syndrome.” The authors also recommended a need for consensus on the definition of PCC. Another scoping review on medications for PCC condition has recently been published and identified 52 relevant studies [38], investigating therapies such as ivabradine, beta‐adrenoreceptor blockers, and local and systemic corticosteroids. However, half of the identified studies were still ongoing, and the vast majority of completed studies were nonexperimental (i.e., case reports/series, observational studies), providing limited evidence on the efficacy of therapy.

### 4.5 Implications for practice

We rated the certainty of evidence as low to very low, downgrading due to study design limitations, inconsistency, and imprecision. Our findings arise from a small number of small studies that include people with PCC defined in different ways. The studies included in this review suggest there may be evidence of improvements in olfactory function for people with PCC treated with oral corticosteroid (methylprednisone) and olfactory training compared with those exposed to olfactory training only and for those receiving oral corticosteroid (prednisone) and nasal irrigation with betamethasone (steroid), abroxol (mucolytic), and rinazine (decongestant) compared with no treatment. However, the evidence is uncertain. In addition, people with PCC exposed to inhaled hydrogen compared with inhaled ambient air may have improved respiratory function, but again the evidence is of low certainty.

### 4.6 Implications for research

We identified a relatively large number of ongoing studies on pharmacological interventions for the treatment of people with PCC. The ongoing studies seek to report outcomes of nervous system functioning, symptoms and conditions, and respiratory functioning, symptoms and conditions. We recommend that future trials use consistent criteria for defining PCC, and we recommend using the definition of PCC provided by the WHO. Larger, high‐quality trials are needed to increase the precision of effect estimates and minimize design limitations. Conducting these as international, multi‐site studies may enhance geographic representativeness. We recommend that trialists also use the available core outcomes set for PCC in deciding which outcomes to measure and report in their trials.

## 5 CONCLUSION

PCC affects relatively large numbers of people worldwide and causes significant negative impacts on health, daily functioning, and ability to work. There is an urgent need to identify effective treatments for PCC and large multi‐site pragmatic trials are required. Currently, there is limited evidence on the effectiveness of pharmacological interventions for the treatment of people with PCC. Published studies are small and of low quality and focus largely on corticosteroids for olfactory dysfunction. However, there is a significant body of research underway that could provide evidence for future pharmacological interventions targeting key clinical outcomes for persons with PCC. It is important that these studies are considered in future guideline updates.

## AUTHOR CONTRIBUTIONS

**K. M. Saif‐Ur‐Rahman**: Conceptualization, data curation, formal analysis, investigation, methodology, project administration, resources, supervision, validation, visualization, writing—original draft, writing—review & editing. **Kavita Kothari**: Data curation, writing—review & editing. **Corinna Sadlier**: Investigation, Methodology, writing—review & editing. **Frank Moriarty**: Investigation, validation, writing—review & editing. **Ani Movsisyan**: Conceptualization, investigation, methodology, validation, writing—review & editing. **Sean Whelan**: Data curation, writing—original draft, writing—review & editing. **Petek E. Taneri**: Data curation, writing—review & editing. **Matthew Blair**: Data curation, writing—original draft, writing—review & editing. **Gordon Guyatt**: Methodology, supervision, validation, writing—review & editing. **Declan Devane**: Conceptualization, formal analysis, funding acquisition, investigation, methodology, project administration, resources, supervision, validation, visualization, writing—review & editing.

## CONFLICT OF INTEREST STATEMENT

The authors declare no conflict of interest.

## Supporting information

Supporting information.

## Data Availability

Available from the corresponding author upon considerable request.
