# Supplementary material for: Effect of pharmacological interventions for the treatment of people with post‐COVID‐19 condition: A rapid review
Source: Cochrane Evid Synth Methods. 2023 Mar 20;1(1):e12001. doi: 10.1002/cesm.12001 (PMC11795952; doi:10.1002/cesm.12001)
Supplement: Supplementary file 1 — Supporting information. [file CESM-1-e12001-s001.docx]

**Supplementary material**

A. The symptoms of post-COVID-19 condition

B. Definitions

C. Search strategy

D. Characteristics of included studies (ongoing)

E. List of excluded studies

F. Risk of bias on included studies

G. Comparison of interventions with steroids versus control

H. Summary of findings - Effect of corticosteroids for the treatment of people with post-COVID-19 condition

**A. The symptoms of post-COVID-19 condition include:**

Abdominal pain

Menstrual and period problems

Altered smell/taste

Anxiety

Blurred vision

Chest pain

Cognitive dysfunction/brain fog

Cough

Depression

Dizziness

Fatigue

Intermittent fever

Gastrointestinal issues (diarrhoea, constipation, acid reflux)

Headache

Memory issues

Joint pain

Muscle pain/spasms

Neuralgias

New onset allergies

Pins and needles sensations

Post-exertional malaise

Shortness of breath

Sleep disorders

Tachycardia/palpitations

Tinnitus and other hearing issues

(Soriano et al. 2022)

**B. Definitions:**

“Fluctuate – A change from time to time in quantity or quality.

Relapse – A return of disease manifestations after a period of improvement.

Cluster – Two or more symptoms that are related to each other and that occur together. They are composed of stable groups of symptoms, are relatively independent of other clusters, and may reveal specific underlying dimensions of symptoms”

(Soriano et al. 2022, page e105).

**C. Search strategy**

Search strategy for Medline (Ovid)

| **#** | **Search** |
| --- | --- |
| 1 | COVID-19/rh |
| 2 | (longcovid* or long covid* or longcoronavirus* or longcorona* virus* or long coronavirus* or long corona* virus* or longCov or long Cov or longsars* or long sars* or "long severe acute respiratory syndrome*" or longncov* or long ncov*).ti,ab,kw,kf. |
| 3 | (("long* term*" or longterm* or "long* haul*" or longhaul* or "long* tail*" or longtail* or longduration* or "long duration*" or longlast* or "long last*" or longstanding* or "long standing*" or "medium* term*" or mediumterm*) adj3 (covid* or coronavirus* or corona* virus* or Cov or "SARS-CoV-2*" or "SARSCoV-2*" or "SARSCoV2*" or "SARS-CoV2*" or "severe acute respiratory syndrome*" or Ncov* or "n-cov")).ti,ab. |
| 4 | ((long* or postacute* or "post acute*" or postdischarg* or "post discharg*" or postinfect* or "post infect*" or postviral* or "post viral*" or postvirus* or "post virus*") adj1 (covid* or coronavirus* or corona* virus* or Cov or "SARS-CoV-2*" or "SARSCoV-2*" or "SARSCoV2*" or "SARS-CoV2*" or "severe acute respiratory syndrome*" or Ncov* or "n-cov")).ti,ab. |
| 5 | ((postcovid* or post covid* or postcoronavirus* or postcorona* virus* or post coronavirus* or post corona* virus* or postCov or post Cov or postsars* or post sars* or "post severe acute respiratory syndrome*" or postncov* or post ncov*) adj3 (syndrome* or disorder* or illness* or sickness* or disease* or condition* or symptom* or sign* or feature* or survivor* or survival* or outpatient* or "out patient*")).ti,ab,kw,kf. |
| 6 | ((ongoing* or endur* or long* or legacy* or slow* or gradual* or protract* or lengthy* or chronic* or persist* or relaps* or remit* or remission* or residual* or delay* or prolong* or extend* or linger* or permanent* or fluctuat* or multisystem* or "multi system*" or nonrecover* or "non recover*" or subacute* or "sub acute*" or lasting* or continuous* or continual* or continuing* or postacute* or "post acute*" or postdischarg* or "post discharg*" or postinfect* or "post infect*" or postviral* or "post viral*" or postvirus* or "post virus*" or "medium* term*" or mediumterm*) adj3 (sequela* or complication* or consequence* or consequent* or complexit* or impair* or problem* or symptom* or disorder* or dysfunction* or manifest*) adj10 (covid* or coronavirus* or corona* virus* or Cov or "SARS-CoV-2*" or "SARSCoV-2*" or "SARSCoV2*" or "SARS-CoV2*" or "severe acute respiratory syndrome*" or Ncov* or "n-cov")).ti,ab. |
| 7 | ((ongoing* or endur* or long* or legacy* or slow* or gradual* or protract* or lengthy* or chronic* or persist* or relaps* or remit* or remission* or residual* or delay* or prolong* or extend* or linger* or permanent* or fluctuat* or multisystem* or "multi system*" or nonrecover* or "non recover*" or subacute* or "sub acute*" or lasting* or continuous* or continual* or continuing* or postacute* or "post acute*" or postdischarg* or "post discharg*" or postinfect* or "post infect*" or postviral* or "post viral*" or postvirus* or "post virus*" or "medium* term*" or mediumterm*) adj3 ((health* or adverse* or dangerous* or harmful* or indirect* or injurious* or secondary* or side* or undesirable* or negative* or damaging* or detriment* or abnormal*) adj3 (effect* or event* or impact* or outcome* or reaction* or aftercare* or issue* or condition*)) adj10 (covid* or coronavirus* or corona* virus* or Cov or "SARS-CoV-2*" or "SARSCoV-2*" or "SARSCoV2*" or "SARS-CoV2*" or "severe acute respiratory syndrome*" or Ncov* or "n-cov")).ti,ab. |
| 8 | or/1-7 |
| 9 | SARS-CoV-2/ or COVID-19/ or (covid* or coronavirus* or corona* virus* or Cov or "SARS-CoV-2*" or "SARSCoV-2*" or "SARSCoV2*" or "SARS-CoV2*" or "severe acute respiratory syndrome*" or Ncov* or "n-cov").ti,kw,kf. |
| 10 | ("long* haul*" or longhaul* or "long* tail*" or longtail* or longduration* or "long duration*" or longlast* or "long last*" or longstanding* or "long standing*").ti,kw,kf. |
| 11 | (postcovid* or post covid* or postcoronavirus* or postcorona* virus* or post coronavirus* or post corona* virus* or postCov or post Cov or postsars* or post sars* or "post severe acute respiratory syndrome*" or postncov* or post ncov* or postacute* or "post acute*" or postdischarg* or "post discharg*" or postinfect* or "post infect*" or postviral* or "post viral*" or postvirus* or "post virus*").ti,kw,kf. |
| 12 | sequela*.ti,ab,kw,kf. |
| 13 | Aftercare/ or rehabilitation/ or convalescence/ or Long Term Adverse Effects/ or Persistent Infection/ |
| 14 | or/10-13 |
| 15 | 9 and 14 |
| 16 | 8 or 15 |
| 17 | Randomized Controlled Trial/ OR Controlled Clinical Trial/ OR Quasi Experimental Study/ OR Pretest Posttest Control Group Design/ OR Time Series Analysis/ OR Experimental Design/ OR Multicenter Study/ OR (randomis* OR randomiz* OR randomly).ti,ab. OR groups.ab.OR (trial OR multicentre OR multicenter OR multi centre OR multi center).ti. OR (intervention? OR effect? OR impact? OR controlled OR control group? OR (before adj5 after) OR (pre adj5 post) OR ((pretest OR pre test) and (posttest OR post test)) OR quasiexperiment* OR quasi experiment* OR pseudo experiment* OR pseudoexperiment* OR evaluat* OR time series OR time point? OR repeated measur*).ti,ab. |
| 18 | 16 and 17 |
| 19 | (letter or historical article or comment or editorial or news or case reports).pt. |
| 20 | 18 NOT 19 |
| 21 | (Animals/ not humans/) |
| 22 | 20 NOT 21 |
| 23 | limit 22 to yr="2020-Current" |

Adapted-from-https://www.nice.org.uk/guidance/ng188/evidence

NICE (2021) Search strategies to support the COVID-19 rapid guideline: managing the long-term effects of COVID-19 (NICE Guideline 188)

Search strategy for EMBASE

| **#** | **Search** |
| --- | --- |
| 1 | coronavirus disease 2019'/exp/dm_rh |
| 2 | (longcovid* OR long-covid* OR longcoronavirus* OR longcorona*-virus* OR long-coronavirus* OR long-corona*-virus* OR longCov OR long-Cov OR longsars* OR long-sars* OR long-severe-acute-respiratory-syndrome* OR longncov* OR long-ncov*):ti,ab,kw |
| 3 | ((long*-term* OR longterm* OR long*-haul* OR longhaul* OR long*-tail* OR longtail* OR longduration* OR long-duration* OR longlast* OR long-last* OR longstanding* OR long-standing* OR medium*-term* OR mediumterm*) NEAR/3 (covid* OR coronavirus* OR corona*-virus* OR Cov OR SARS-CoV-2* OR SARSCoV-2* OR SARSCoV2* OR SARS-CoV2* OR severe-acute-respiratory-syndrome* OR Ncov* OR n-cov)):ti,ab |
| 4 | ((long* OR postacute* OR post-acute* OR postdischarg* OR post-discharg* OR postinfect* OR post-infect* OR postviral* OR post-viral* OR postvirus* OR post-virus*) NEAR/1 (covid* OR coronavirus* OR corona*-virus* OR Cov OR SARS-CoV-2* OR SARSCoV-2* OR SARSCoV2* OR SARS-CoV2* OR severe-acute-respiratory-syndrome* OR Ncov* OR n-cov)):ti,ab |
| 5 | ((postcovid* OR post-covid* OR postcoronavirus* OR postcorona*-virus* OR post-coronavirus* OR post-corona*-virus* OR postCov OR post-Cov OR postsars* OR post-sars* OR post-severe-acute-respiratory-syndrome* OR postncov* OR post-ncov*) NEAR/3 (syndrome* OR disorder* OR illness* OR sickness* OR disease* OR condition* OR symptom* OR sign* OR feature* OR survivor* OR survival* OR outpatient* OR out-patient*)):ti,ab,kw |
| 6 | ((ongoing* OR endur* OR long* OR legacy* OR slow* OR gradual* OR protract* OR lengthy* OR chronic* OR persist* OR relaps* OR remit* OR remission* OR residual* OR delay* OR prolong* OR extend* OR linger* OR permanent* OR fluctuat* OR multisystem* OR multi-system* OR nonrecover* OR non-recover* OR subacute* OR sub-acute* OR lasting* OR continuous* OR continual* OR continuing* OR postacute* OR post-acute* OR postdischarg* OR post-discharg* OR postinfect* OR post-infect* OR postviral* OR post-viral* OR postvirus* OR post-virus* OR medium*-term* OR mediumterm*) NEAR/3 (sequela* OR complication* OR consequence* OR consequent* OR complexit* OR impair* OR problem* OR symptom* OR disorder* OR dysfunction* OR manifest*) NEAR/10 (covid* OR coronavirus* OR corona*-virus* OR Cov OR SARS-CoV-2* OR SARSCoV-2* OR SARSCoV2* OR SARS-CoV2* OR severe-acute-respiratory-syndrome* OR Ncov* OR n-cov)):ti,ab |
| 7 | ((ongoing* OR endur* OR long* OR legacy* OR slow* OR gradual* OR protract* OR lengthy* OR chronic* OR persist* OR relaps* OR remit* OR remission* OR residual* OR delay* OR prolong* OR extend* OR linger* OR permanent* OR fluctuat* OR multisystem* OR multi-system* OR nonrecover* OR non-recover* OR subacute* OR sub-acute* OR lasting* OR continuous* OR continual* OR continuing* OR postacute* OR post-acute* OR postdischarg* OR post-discharg* OR postinfect* OR post-infect* OR postviral* OR post-viral* OR postvirus* OR post-virus* OR medium*-term* OR mediumterm*) NEAR/3 (health* OR adverse* OR dangerous* OR harmful* OR indirect* OR injurious* OR secondary* OR side* OR undesirable* OR negative* OR damaging* OR detriment* OR abnormal*) NEAR/3 (effect* OR event* OR impact* OR outcome* OR reaction* OR aftercare* OR issue* OR condition*) NEAR/10 (covid* OR coronavirus* OR corona*-virus* OR Cov OR SARS-CoV-2* OR SARSCoV-2* OR SARSCoV2* OR SARS-CoV2* OR severe-acute-respiratory-syndrome* OR Ncov* OR n-cov)):ti,ab |
| 8 | #1 OR #2 OR #3 OR #4 OR #5 OR #6 OR #7 |
| 9 | Severe acute respiratory syndrome coronavirus 2'/exp OR 'coronavirus disease 2019'/exp OR (covid* OR coronavirus* OR corona*-virus* OR Cov OR SARS-CoV-2* OR SARSCoV-2* OR SARSCoV2* OR SARS-CoV2* OR severe-acute-respiratory-syndrome* OR Ncov* OR n-cov):ti,kw,de |
| 10 | (long*-haul* OR longhaul* OR long*-tail* OR longtail* OR longduration* OR long-duration* OR longlast* OR long-last* OR longstanding* OR long-standing*):ti,kw,de |
| 11 | (postcovid* OR post-covid* OR postcoronavirus* OR postcorona*-virus* OR post-coronavirus* OR post-corona*-virus* OR postCov OR post-Cov OR postsars* OR post-sars* OR post-severe-acute-respiratory-syndrome* OR postncov* OR post-ncov* OR postacute* OR post-acute* OR postdischarg* OR post-discharg* OR postinfect* OR post-infect* OR postviral* OR post-viral* OR postvirus* OR post-virus*):ti,kw,de |
| 12 | sequela*:ti,ab,kw,de |
| 13 | aftercare'/de OR 'rehabilitation'/exp OR 'convalescence'/exp or 'persistent virus infection'/exp |
| 14 | #10 OR #11 OR #12 OR #13 |
| 15 | #9 AND #14 |
| 16 | #8 OR #15 |
| 17 | randomized controlled trial'/exp OR 'controlled clinical trial'/exp OR 'quasi experimental study'/exp OR 'pretest posttest control group design'/exp OR 'time series analysis'/exp OR 'experimental design'/exp OR 'multicenter study'/exp OR (randomis* OR randomiz* OR randomly):ti,ab OR groups:ab OR (trial OR multicentre OR multicenter OR multi-centre OR multi-center):ti OR (intervention? OR effect? OR impact? OR controlled OR control-group? OR (before NEAR/5 after) OR (pre NEAR/5 post) OR ((pretest OR pre-test) and (posttest OR post-test)) OR quasiexperiment* OR quasi-experiment* OR pseudo-experiment* OR pseudoexperiment* OR evaluat* OR time-series OR time-point? OR repeated-measur*):ti,ab |
| 18 | #16 AND #17 |
| 19 | [letter]/lim OR [conference abstract]/lim OR [conference paper]/lim OR [conference review]/lim OR [editorial]/lim OR [note]/lim |
| 20 | [animals]/lim NOT [humans]/lim |
| 21 | #18 NOT #19 NOT #20 |

Adapted-from-https://www.nice.org.uk/guidance/ng188/evidence

NICE (2021) Search strategies to support the COVID-19 rapid guideline: managing the long-term effects of COVID-19 (NICE Guideline 188)

Search strategy for ClinicalTrials.gov

| **#** | **Search (more specific) Title only** |
| --- | --- |
| 1 | "PCC" OR "Rehab" OR "rehabilitation" OR "Fatigue" OR "Long covid" OR "long covid 19" OR "sequelae" OR "lingering" OR "prolong" OR "prolonged" OR "long-covid" OR "long-covid19" OR "longcovid" OR "longcovid19" OR "longer" OR "after convelescence" |
| 2 | "postcovid" OR "postcovid19" OR "post covid" OR "post covid19" OR "post-covid" OR "post-covid19" OR "post coronavirus" OR "post-coronavirus" OR "postacute" OR "post acute" OR "post-acute" OR "post-ARDS" |
| 3 | "post infection" OR "post-infection" OR "post-inpatient" OR "post sars-cov-2" OR "post sars cov 2" OR "long hauler" OR "long haulers" OR "longhauler" OR "longhaulers" OR "long-hauler" OR "long-haulers" |
| 4 | "longstanding" OR "medium-term" OR "medium term" OR "postviral" OR "post viral" OR "post-viral" OR "postdischarge" OR "post-discharge" OR "post intensive" OR "post-intensive" |
| 5 | \| Active, not recruiting, Completed Studies |

Search strategy for International Clinical Trials Registry Platform (ICTRP)

| **#** | **Search** |
| --- | --- |
| Title Contains | PCC |
|  | Rehab |
|  | Fatigue |
|  | Long covid |
|  | sequelae |
|  | lingering |
|  | prolong |
|  | long-covid |
|  | longcovid |
|  | longer |
|  | after convelescen |
|  | postcovid |
|  | post covid |
|  | post-covid |
|  | post corona |
|  | post-corona |
|  | postacute |
|  | post acute |
|  | post-acute |
|  | post-ARDS |
|  | post infection |
|  | post-infection |
|  | post-inpatient |
|  | post sars-cov |
|  | long hauler |
|  | longhauler |
|  | long-hauler |
|  | longstandng |
|  | medium-term |
|  | postviral |
|  | post viral |
|  | post-viral |
|  | postdischarge |
|  | post-discharge |
|  | post intensive |
|  | post-intensive |

**D: Characteristics of included studies (ongoing)**

| **Trial registration number** | **Population age and gender** | **Intervention and control** | **Review outcome (actual outcome used in study)** | **Study design** | **Sample size** | **Starting date and anticipated completion date** |
| --- | --- | --- | --- | --- | --- | --- |
| NCT04695704 | Age range 18 to 80 years; both male and female | Intervention: Montelukast 10 mg  Control: Placebo (Composition not reported) | Respiratory functioning, symptoms and conditions (health-related quality of life associated with respiratory symptoms) | Randomised placebo-controlled trial (participant, care provider, investigator, outcomes assessor blinded) | Intervention 142  Control 142 | Starting date: August 2021  Completion date: March 2023 |
| NCT05242003 | 18 years and older; both male and female | Intervention: Immuno-metabolic Regulator;(600mg MYMD-1 and 300mg MYMD-1)  Control: Placebo (Composition not reported) | Mental functioning, symptoms and conditions (change in depressive symptoms as assessed by the Montgomery-Asberg Depression Rating Scale (MADRS) | Randomised placebo-controlled trial (participant, investigator blinded) | 90 participants | Starting date: March 2022  Completion date: Not mentioned |
| CTRI/2021/12/038577 | Age range 18 to 80 years; both male and female | Intervention: Deupirfenidone Capsules - (LYT-100) 250 mg  Control: Placebo (Composition not reported) | Respiratory functioning, symptoms and conditions (change in distance walked on six-minute walk test - 6MWT) | Randomised placebo-controlled trial (participant, investigator blinded) | 168 participants | Starting date: December 2021  Completion date: Not mentioned |
| NCT04592354 | Age range 18 to 65 years; female only | Intervention: Anhydrous enol-oxaloacetate 500 mg  Control: Placebo (Composition not reported) | Fatigue or Exhaustion (Fatigue with the Fatigue Severity Scale (FSS));  Mental functioning, symptoms and conditions (Depression with Beck's Depression Inventory (BDI)) | Randomised placebo-controlled trial (participant, care provider, investigator, outcomes assessor blinded) | 40 participants | Starting date: October 2020  Completion date: June 2021 |
| NCT04374474 | 18 years and older; both male and female | Intervention: Nasal irrigation with budesonise and olfactory training  Control: Olfactory training | Nervous system functioning, symptoms and conditions (change in Snap and Sniff Threshold Test and Smell Identification Test) | Open labelled randomised placebo-controlled trial | Not mentioned | Starting date: January 2021  Completion date: March 2022 |
| NCT04880161 | 18 years and older; both male and female | Intervention: Inhaled Ampion (low molecular weight filtrate of human serum albumin)  Control: Placebo (Composition not reported) | Respiratory functioning, symptoms and conditions (respiratory Symptoms) | Randomised placebo-controlled trial (participant, care provider, investigator, outcomes assessor blinded) | 32 participants | Starting date: July 2021  Completion date: February 2022 |
| CTRI/2020/11/029305 | Age range 18 to 65 years; both male and female | Intervention: Oral clarithromycin 500 mg  Control: No intervention | Respiratory functioning, symptoms and conditions (resolution of lung findings in Pneumonia) | Open labelled randomised controlled trial | 100 participants | Starting date: November 2020  Completion date: Not mentioned |
| NCT04657809 | Age range 18 to 70 years; both male and female | Intervention: Formulated bioadhesive fast dissolving film containing 100 IU of insulin  Control: Formulated bioadhesive fast dissolving film containing no drug | Nervous system functioning, symptoms and conditions (smell sensation improvement) | Randomised placebo-controlled trial (participant, care provider blinded) | 40 participants | Starting date: October 2020  Completion date: March 2021 |
| NCT04818489 | 18 years and older; both male and female | Intervention: Colchicine 0.5 mg (2 tablets: 1 mg)  Control: No intervention | Respiratory functioning, symptoms and conditions (clinical status; pulmonary fibrosis) | Randomised controlled trial (outcome assessor blinded) | 260 participants | Starting date: March 2021  Completion date: October 2021 |
| CTRI/2021/11/038234 | 18 years and older; both male and female | Intervention: Colchicine 0.5 mg tablets  Control: Placebo (Composition not reported) | Respiratory functioning, symptoms and conditions (change in 6-minute walk test) | Randomised placebo-controlled trial (participant, investigator, blinded) | 350 participants | Starting date: December 2021  Completion date: Not mentioned |
| NCT04657484 | 18 years and older; both male and female | Intervention: Medium dose prednisolone  Control: Low dose prednisolone | Respiratory functioning, symptoms and conditions (radiologic response) | Open labelled randomised controlled trial | 130 participants | Starting date: December 2020  Completion date: September 2021 |
| NCT04678830 | 18 years and older; both male and female | Intervention: Leronlimab (PRO) 140 - a humanized IgG4, monoclonal antibody  Control: Placebo (Composition not reported) | Physical functioning, symptoms and conditions (COVID-19-related symptom severity score) | Randomised placebo-controlled trial (participant, care provider, investigator, outcomes assessor blinded) | 56 participants | Starting date: March 2021  Completion date: July 2021 |
| NCT05184192 | Age range 18 to 65 years; both male and female | Intervention: Gabapentin gelatin capsules 300mg  Control: Placebo (lactose monohydrate NF) | Nervous system functioning, symptoms and conditions (Clinical Global Impression of Improvement Scale (CGI-I); University of Pennsylvania Smell Identification Test) | Randomised placebo-controlled trial (participant, investigator, outcomes assessor blinded) | 50 participants | Starting date: January 2022  Completion date: April 2022 |
| NCT05216614 | Age range 18 to 70 years; both male and female | Intervention: Fluvoxamine  Control: Placebo (Composition not reported)  lactose | Nervous system functioning, symptoms and conditions (Clinical Global Impression Scale (CGI)) | Randomised placebo-controlled trial (participant, investigator, outcomes assessor blinded) | Not mentioned | Starting date: December 2021  Completion date: February 2022 |
| NCT04801940 | 18 years and older; both male and female | Intervention: Apixaban 2.5mg; Atorvastatin 40mg  Control: Usual post-hospital care. | Survival (Hospital free survival; All-cause mortality) | Open labelled randomised controlled trial | 2631 participants | Starting date: May 2021  Completion date: January 2024 |
| NCT04905888 | Age range 18 to 65 years; both male and female | Intervention: Hyperbaric oxygen therapy  Control: No intervention | Respiratory functioning, symptoms and conditions (exercise tolerance) | Open labelled randomised controlled trial | 24 participants | Starting date: November 2021  Completion date: December 2023 |
| NCT04647656 | 18 years and older; both male and female | Intervention: Hyperbaric oxygen therapy  Control: Non hyberboric oxygen | Cognitive functioning, symptoms and conditions (cognitive health assessment) | Randomised placebo-controlled trial (participant, care provider, investigator, outcomes assessor blinded) | 91 participants | Starting date: December 2020  Completion date: January 2022 |
| NCT05350774 | 18 years and older; both male and female | Intervention arm 1: IV immunoglobulin 0.4g/kg/day; Intervention arm 2: IV methylprednisolone 1g/day  Control: IV normal saline 250ml | Nervous system functioning, symptoms and conditions (neurological Post-Acute Sequelae) | Randomised placebo-controlled trial (participant, care provider, investigator, blinded) | 60 participants | Starting date: May 2022  Completion date: January 2022 |
| ACTRN12621000637842 | 18 years and older; both male and female | Intervention: Colchicine 0.5 mg tablet  Control: Standard care | Respiratory functioning, symptoms and conditions (Maximum oxygen requirement) | Open labelled randomised controlled trial | 1000 participants | Starting date: August 2021  Completion date: February 2023 |
| NCT05274477 | Age range 18 to 60 years; both male and female | Intervention: Fampridine SR 10 mg  Control: Placebo (Composition not reported) | Cognitive functioning, symptoms and conditions (Digits Span backward performance) | Randomised placebo-controlled trial (participant, care provider, investigator, outcomes assessor blinded) | 44 participants | Starting date: June 2022  Completion date: May 2023 |
| RPCEC00000376 | 18 years and older; both male and female | Intervention: 200,000 IU of ior®EPOCIM intravenously  Control: Supportive treatment | Cardiovascular functioning, symptoms and conditions (cardiovascular, renal) and/or Respiratory functioning, symptoms and conditions (respiratory disorders) | Open labelled randomised controlled trial | 135 participants | Starting date: Not mentioned  Completion date: December 2023 |
| NCT05269030 | 18 years and older; both male and female | Intervention: Topical Ivermectin 1% nasal drop  Control: Local budesonide 64 µg/puff nasal spray. | Nervous system functioning, symptoms and conditions (Parosmia) | Randomised controlled trial (outcome assessor-blinded) | 60 participants | Starting date: July 2022  Completion date: March 2023 |
| NCT04534478 | 18 years and older; both male and female | Intervention: Prednisone 0.5mg/kg/day for 3 weeks; 20mg/day for 3 weeks; 15mg/day for 2 weeks; 10mg/day for 2 weeks, 5mg/day for 2 weeks and discontinue.  Control: Prednisone 0.75mg/kg/day for 4 weeks; 0.5mg/kg/day for 4 weeks, 20mg/day for 4 weeks; 10mg/day for 6 weeks, 5mg/day for 6 weeks. | Respiratory functioning, symptoms and conditions (Change in pulmonary diffusion) | Open labelled randomised controlled trial | 120 participants | Starting date: September 2020  Completion date: December 2021 |
| NCT04944121 | Age range 18 to 75 years; both male and female | Intervention: RSLV-132 (an enzymatically active ribonuclease biologic drug)  10 mg/kg IV  Control: Placebo (Composition not reported)  Sodium chloride 0.9% | Fatigue or Exhaustion (PROMIS Fatigue SF 7a T-score; FACIT Fatigue questionnaire; Long COVID-19-related Symptom Assessment patient questionnaire; Patient-reported Global Impression of Severity questionnaire; Digit Symbol Substitution Test; Physician Global Assessment) | Randomised placebo-controlled trial (participant, care provider, investigator, outcomes assessor blinded) | 70 participants | Starting date: June 2021  Completion date: March 2023 |
| NCT04527354 | Age range 18 to 75 years; both male and female | Intervention: Treamid 50 mg  Control: Placebo (Composition not reported) | Respiratory functioning, symptoms and conditions (Clinically significant change in FVC and/or DLCO at) | Randomised placebo-controlled trial (participant, investigator blinded) | 60 participants | Starting date: September 2020  Completion date: February 2021 |
| NCT04607928 | 18 years and older; both male and female | Intervention: Pirfenidone  Control: Placebo (Composition not reported) | Respiratory functioning, symptoms and conditions (Pulmonary Fibrosis) | Randomised placebo-controlled trial (participant, care provider, investigator blinded) | 148 participants | Starting date: August 2020  Completion date: June 2022 |
| CTRI/2021/09/036442 | Age range 18 to 80 years; both male and female | Intervention: Pirfenidone 267mg tablets  Control: Pulmonary rehabilitation | Respiratory functioning, symptoms and conditions (Pulmonary Fibrosis) | Open labelled randomised controlled trial | 60 participants | Starting date: September 2021  Completion date: Not mentioned |
| UMIN000043537 | Age range 18 to 65 years; both male and female | Intervention: Combination of local corticosteroids and antihistamines nasal spray  Control: Normal saline 0.2% | Nervous system functioning, symptoms and conditions (Anosmia) | Randomised placebo-controlled trial (participant, care provider, investigator, outcomes assessor blinded) | 200 participants | Starting date: January 2021  Completion date: Not mentioned |
| NCT05212831 | 18 years and older; both male and female | Intervention: Portable Oxygen Concentrator  Inogen One® G4  Control: Standard of Care | Nervous system functioning, symptoms and conditions (Brain hypoxia and peripheral oxygen saturation); Cardiovascular functioning, symptoms and conditions (VO2max during cardiopulmonary exercise test); Cognitive functioning, symptoms and conditions (MoCA test; Other neuropsychological tests) | Open labelled randomised controlled trial | 20 participants | Starting date: August 2022  Completion date: December 2022 |
| NCT04978259 | 18 years and older; both male and female | Intervention: Intravenous remdesivir  Control: Standard of Care | Physical functioning, symptoms and conditions (EQ-VAS; EQ-5D-5L); Recovery (Recovery); Fatigue or Exhaustion (Fatigue; Exertional dyspnea) | Open labelled randomised controlled trial | 202 participants | Starting date: July 2021  Completion date: December 2023 |
| NCT04951362 | Age range 18 to 70 years; both male and female | Intervention: Ivermectin intranasal spray  Control: Saline nasal spray | Nervous system functioning, symptoms and conditions (Regaining of smell) | Open labelled randomised controlled trial | Intervention: 49;  Control: 47 | Starting date: April 2021  Completion date: May 2022 |
| NCT04842448 | Age range 18 to 60 years; both male and female | Intervention: Hyperbaric oxygen 240 kPa  Control: Sham treatment 134-120 kPa Air | Respiratory functioning, symptoms and conditions (Endothelial dysfunction; 6-min walk test; 30/60 min chair stand; EuroQol-5 Dimensions questionnaire; RAND 36 normalization) | Randomised controlled trial (outcome assessor-blinded) | 80 participants | Starting date: September 2021  Completion date: December 2023 |
| NCT04949386 | Age range 18 to 80 year; both male and female | Intervention: perfluorooctylbromide (PFOB) delivered in gas/aerosol/vapor form within a medical gas mixture containing 8% CO_2_.  Control: Placebo (medical grade air with 3ml saline (0.9% NaCl)) | Respiratory functioning, symptoms and conditions (Normalization in 6-minute walk test (6MWT) distance; Changes in per cent predicted forced expiratory volume in 1 second; Changes from baseline in respiratory symptoms assessed by the St George's Respiratory Questionnaire (SGRQ)) | Randomised placebo-controlled trial (participant, care provider, investigator, outcomes assessor blinded) | 48 participants | Starting date: September 2021  Completion date: January 2022 |
| NCT04551781 | 18 years and older; both male and female | Intervention: Prednisolone 20 mg  Control: Placebo (Composition not reported) | Respiratory functioning, symptoms and conditions (Pulmonary Fibrosis, resolution of CT chest infiltrates) | Randomised controlled trial (outcome assessor-blinded) | 450 participants | Starting date: April 2020  Completion date: July 2020 |
| NCT05220280 | 18 years and older; both male and female | Intervention: Oral imatinib 400 mg tablet; Infliximab single IV infusion  Control: Standard of care | Physical functioning, symptoms and conditions (Long-COVID symptoms; Health-related quality of life (EQ-5D-5L questionnaire)); Mortality; Incidence of comorbidity; Respiratory functioning, symptoms and conditions (Lung function) | Open labelled randomised controlled trial | 400 participants | Starting date: February 2020  Completion date: December 2025 |
| NCT04705831 | Age range 18 to 75 year; both male and female | Intervention: Ruconest (intravenous)  C1 Esterase Inhibitor  Control: Placebo (Composition not reported) | Nervous system functioning, symptoms and conditions; Cognitive functioning, symptoms and conditions (Neuropsychological Measures (BRIEF-A); Neuropsychological Measures (RBANS); Neuropsychological Measures (BDI II); Neuropsychological Measures (MoCA); Patient-Rate Questionnaires (FSS); Patient-Rate Questionnaires (MIDAS)) | Randomised placebo-controlled trial (participant, care provider blinded) | 40 participants | Starting date: December 2020  Completion date: January 2022 |
| NCT04988282 | 18 years and older; both male and female | Intervention: Methylprednisolone, 0.5 mg/kg/day  Control: Standard of care | Respiratory functioning, symptoms and conditions (Improvement of diffusion capacity of the lung for carbon monoxide (DLCO); Improvement of Forced Vital Capacity (FVC); Improvement of arterial oxygen saturation (SaO2); Improvement of Exercise Capacity) | Open labelled randomised controlled trial | 642 participants | Starting date: May 2021  Completion date: December 2021 |
| NCT05104424 | Age range 18 to 75 year; both male and female | Intervention: Intranasal rapid insulin 40 IU as 0.1 ml  Control: Placebo (patients taking zinc only with smell training on volatile oils) | Nervous system functioning, symptoms and conditions (Evaluation of disturbances of smell and taste (Sniffin 'Sticks" test); evaluation of taste disorders; questionnaire for taste self-assessment ( Dynachron-olfaction questionnaire)) | Open labelled randomised controlled trial | Intervention: 22  Control: 22 | Starting date: December 2021  Completion date: October 2022 |
| NCT05269017 | 18 years and older; both male and female | Intervention: Vitamin D nasal drops  Control: Budesonide 64 µg/puff nasal spray | Nervous system functioning, symptoms and conditions (Parosmia) | Randomised controlled trial (outcome assessor-blinded) | 60 participants | Starting date: June 2022  Completion date: March 2023 |
| NCT05047952 | 18 years and older; both male and female | Intervention: Vortioxetine  Control: Placebo (Composition not reported) | Cognitive functioning, symptoms, and conditions )Digit Symbol Substitution Test (DSST); CogState Online Cognitive Battery; Trails Making Test (TMT)-A/B; Rey's auditory verbal learning test (RAVLT); Perceived Deficits Questionnaire, 20-item (PDQ-20)); Fatigue or Exhaustion (Fatigue Severity Scale (FSS)); Mental functioning, symptoms and conditions (Snaith Hamilton Pleasure Rating Scale (SHAPS); Patient Health Questionnaire, 9-item (PHQ-9); Generalized Anxiety Scale, 7-item (GAD-7); World Health Organization Wellbeing Scale, 5-item (WHO-5); EuroQol, 5-dimension, 5-level (EQ-5D-5L); Sheehan Disability Scale (SDS)); Physical functioning, symptoms and conditions (Post-Covid Functional Scale (PCFS)) | Randomised placebo-controlled trial (participant, care provider, investigator, outcomes assessor blinded) | 200 participants | Starting date: September 2021  Completion date: September 2022 |
| NCT05228899 | 18 years and older; both male and female | Intervention: 1 mL of Zofin diluted with 100ml of sterile saline  Control: 1mL of placebo diluted with 100ml of sterile saline | Fatigue or Exhaustion; Mental functioning, symptoms and conditions (Fatigue Severity Score, Beck Depression Inventory Score, Mental Fatigue Questionnaire Score) | Randomised placebo-controlled trial (participant, care provider, investigator blinded) | Intervention: 15  Control: 15 | Starting date: April 2022  Completion date: December 2023 |

**E. List of excluded studies**

**List of excluded studies: (n = 26)**

| **Serial No.** | **Author and publication year** | **Title** | **Reasons for exclusion** |
| --- | --- | --- | --- |
| 1 | Rathi et al, 2021 | A Randomized Controlled Trial of the Efficacy of Systemic Enzymes and Probiotics in the Resolution of Post-COVID Fatigue | Dietary supplements |
| 2 | Pugliese ME et al, 2021 | Anti-SARS-CoV-2 S-RBD IgG Antibody Responses after  COVID-19 mRNA Vaccine in the Chronic Disorder of  Consciousness: A Pilot Study | Not on PCC |
| 3 | Barros CMSS et al, 2021 | Short-Course of Methylprednisolone Improves Respiratory Functional Parameters After 120 Days in Hospitalized COVID-19 Patients (Metcovid Trial): A Randomized Clinical Trial | Not on PCC |
| 4 | Goel N et al, 2021 | Systemic corticosteroids for management of 'long-COVID': an evaluation after 3 months of treatment | Study design |
| 5 | Utrero-Rico A et al, 2021 | A Short Corticosteroid Course Reduces Symptoms and Immunological Alterations Underlying Long-COVID | Study design |
| 6 | Steffens et al, 2022 | Effectiveness and safety of PRP on persistent olfactory dysfunction related to COVID19: towards a new therapeutic hope | Plasma therapy |
| 7 | Tirelli U et al, 2021 | Fatigue in post-acute sequelae of SARS-CoV2 (PASC) treated with oxygen-ozone autohemotherapy – preliminary results on 100 patients | Study design |
| 8 | Santinelli L et al, 2022 | Oral Bacteriotherapy Reduces the Occurrence of Chronic Fatigue in COVID-19 Patients | Not on PCC |
| 9 | Tsuchida T et al, 2022 | Relationship between changes in symptoms and antibody titers after a single vaccination in patients with Long COVID | Study design |
| 10 | Ramacciotti E et al, 2022 | Rivaroxaban versus no anticoagulation for post-discharge thromboprophylaxis after hospitalisation for COVID-19 (MICHELLE): an open-label, multicentre, randomised, controlled trial | Not on PCC |
| 11 | Karosanidze I et al, 2022 | Efficacy of Adaptogens in Patients with Long COVID-19: A Randomized, Quadruple-Blind, Placebo-Controlled Trial | Herbal medicine |
| 12 | Haroon S et al, 2022 | Therapies for Long COVID in nonhospitalised individuals: from symptoms, patient-reported outcomes and immunology to targeted therapies (The TLC Study) | Study design |
| 13 | Chen Y et al, 2022 | Efficacy and safety of Bufei Huoxue capsules in the management of convalescent patients with COVID-19 infection: A multicentre, double-blind, and randomised controlled trial | Traditional Chinese medicine |
| 14 | Cesarone MR et al, 2022 | Pycnogenol®-Centellicum® supplementation improves lung fibrosis and post-COVID-19 lung healing | Herbal medicine |
| 15 | Martinuzzi E et al, 2022 | A Single Dose of BNT162b2 mRNA Vaccine Induces Airway Immunity in SARS-CoV 2 2 Naive and recovered COVID-19 subjects | Not on PCC |
| 16 | Hoque A et al, 2021 | Third dose vaccine With BNT162b2 and its response on Long COVID after Breakthrough infections | Study design |
| 17 | Sing CV et al, 2021 | The outcome of fluticasone nasal spray on anosmia and triamcinolone oral paste in dysgeusia in COVID-19 patients☆ | Not on PCC |
| 18 | Di Stadio A et al, 2022 | Ultramicronized Palmitoylethanolamide and Luteolin Supplement Combined with Olfactory Training to Treat Post-COVID-19 Olfactory Impairment: A Multi-Center Double-Blinded Randomized Placebo-Controlled Clinical Trial | Dietary supplements |
| 19 | D'Ascanio L et al 2021 | Randomized clinical trial “olfactory dysfunction after COVID-19: olfactory rehabilitation therapy vs. intervention treatment with Palmitoylethanolamide and Luteolin”: preliminary results | Dietary supplements |
| 20 | Hernandez AK et al, 2022 | Omega-3 supplementation in postviral olfactory dysfunction: a pilot study | Dietary supplements |
| 21 | Turova et al, 2021 | Hyperbaric oxygenation in outpatient rehabilitation of COVID-19 convalescents | Article written in Russian |
| 22 | Shogenova et al, 2021 | KalHydrogen inhalation in rehabilitation program of the medical staff recovered from COVID-19 | Article written in Russian |
| 23 | Zolotovskaia 2021 | Post-COVID-19 asthenic syndrome | Article written in Russian |
| 24 | Putilina et al, 2021 | The result of prospective randomized study CITADEL - the efficacy and safety of drug cytoflavin in postcovid rehabilitation | Article written in Russian |
| 25 | Gromova et al, 2021 | On the prospects for the use of thiamine, pyridoxine, and cyanocobalamin in the complex therapy and rehabilitation of patients with COVID-19 | Article written in Russian |
| 26 | Bogolepova et al, 2021 | Fatigue and cognitive impairment in post-COVID syndrome: possible treatment approaches | Article written in Russian |

**Reasons for exclusion:**

Not on Post COVID Condition (PCC) = 6, Dietary supplements =4, Traditional Chinese Medicine/ Herbal medicine = 3, Study design = 6, Plasma therapy = 1, Article written in language other than English = 6

**List of excluded trial registry studies (n =38)**

| **Serial No** | **Title and registration number** | **Reasons for exclusion** |
| --- | --- | --- |
| 1 | Efficacy of the combination of modern medicine and traditional Chinese medicine in pulmonary fibrosis arising as a sequela in convalescent COVID-19 patients:  a randomized multicenter trial (ChiCTR2000033284) | Traditional Chinese Medicine |
| 2 | A Phase 2 Randomized, Double-blind, Placebo-controlled Trial and Open Label Extension to Evaluate the Safety and Efficacy of Deupirfenidone (LYT- 100) in Post-acute COVID-19 respiratory disease | Not on PCC |
| 3 | A Randomized, Double Blind, Placebo-Controlled Study to Evaluate the Effect of Nicotinamide Mononucleotide (NMN) As an Adjuvant to Standard of Care (SOC) On Fatigue Associated with COVID-19 Infection (CTRI/2022/02/040399) | Not on PCC |
| 4 | Can ProGenX resolve Post-COVID Fatigue and improve Immune Status? (CTRI/2022/04/041704) | Ayurvedic medicine |
| 5 | Cerebrolycin for Treatment of Covid-related Anosmia and Ageusia (NCT04830943) | Study design |
| 6 | Clinical study of Mulmina Mango as a Health Supplement in Management of Post-COVID-19 () | Ayurvedic medicine |
| 7 | Clinical trial of CIM-Meg19 to combat the severity of disorders in COVID-19 and post COVID-19 patients (CTRI/2021/05/033472) | Ayurvedic medicine |
| 8 | Clinical trial on Post covid fibrosis and idiopathic pulmonary fibrosis | Ayurvedic medicine |
| 9 | Efficacy, Safety, Tolerability of AXA1125 in Fatigue After COVID-19 Infection | Dietary Supplement |
| 10 | Feasibility Pilot Clinical Trial of Omega-3 Supplement vs. Placebo for Post Covid-19 Recovery Among Health Care Workers (NCT05121766) | Dietary Supplement |
| 11 | Diode Laser 940 nm in Management of Loss of Taste Sensation | Phototherapy |
| 12 | Direct Topical Lung T3 Treatment to Improve Outcome & Sequelae of COVID-19 acute respiratory distress syndrome (NCT04725110) | Not on PCC |
| 13 | Effects of Sodium Pyruvate Nasal Spray in COVID-19 Long Haulers (NCT04871815) | Study design |
| 14 | ExoFlo™ Infusion for Post-Acute COVID-19 and Chronic Post-COVID-19 Syndrome | Stem cell therapy |
| 15 | Feasibility of Cannabidiol for the Treatment of Long COVID | Study design |
| 16 | Impact of Monoclonal Antibody Treatment on Post-Acute COVID-19 Syndrome | Study design |
| 17 | Impact of post-Acute respiratory distress syndrome COVID sedation on late neuroinflammation (2020-004802-70) | Study design |
| 18 | Impact of Post-ARDS Covid-19 Sedation on Persistent Neuroinflammation (NCT05233605) | Study design |
| 19 | Intermediate Size Expanded Access Protocol Evaluating HB-adMSC's for the Treatment of Post-COVID-19 Syndrome | Stem cell therapy |
| 20 | Lactoferrin in the treatment of Long COVID (Trial NL9742) | Dietary Supplement |
| 21 | LYT-100 in Post-acute COVID-19 respiratory disease (NCT04652518) | Not on PCC |
| 22 | Nebulized PL for Post-COVID-19 Syndrome | Platelet therapy |
| 23 | Ozone Plasma on Lung Function and Inflammatory Parameters in Pulmonary Sequelae Associated with Coronavirus 19 Infection (NCT05089305) | Ozone Plasma |
| 24 | Pilot Study into LDN and NAD+ for Treatment of Patients with Post-COVID-19 Syndrome (NCT04604704) | Study design |
| 25 | Post-Acute Sequelae of Coronavirus-19 (COVID-19) With Dyspnea on Exertion and Associated TaChycardia TrEatment Study (NCT05096884) | Study design |
| 26 | Randomized Double-Blind Phase 2 Study of Allogeneic HB-adMSCs for the Treatment of Chronic Post-COVID-19 Syndrome | Stem cell therapy |
| 27 | Safety study of health food containing 5-aminolevulinic acid phosphate in patients with COVID-19 sequelae using smart watch and electronic research collaborator diary | Dietary Supplement |
| 28 | Safety and tolerability evaluation of PE in patients with Post-Acute Covid-19 Syndrome (PCC) compared to sham plasma exchange (EudraCT Number: 2022-000641-33) | Plasma therapy |
| 29 | Spa Rehabilitation, Antioxidant and Bioenergetic Supportive Treatment of Patients with Post-Covid-19 Syndrome | Dietary Supplement |
| 30 | Study of Allogeneic Adipose-Derived Mesenchymal Stem Cells to Treat Post COVID-19 "Long Haul" Pulmonary Compromise | Stem cell therapy |
| 31 | Study to Evaluate the Effect of Nicotinamide Mononucleotide (NMN) As an Adjuvant to Standard of Care (SOC) On Fatigue Associated With COVID-19 Infection (NCT05175768) | Not on PCC |
| 32 | Study to investigate the safety and efficacy of the health supplements ImmunoSEB and ProbioSEB CSC3 on patients suffering from COVID-19 Induced Fatigue/Post Viral Fatigue Syndrome (CTRI/2021/05/033576) | Dietary Supplement |
| 33 | WHO COVID-19 - Evaluation of the Efficacy of Probiotics to Reduce the Occurrence of Long COVID (PROVID-LD) (NCT05080244) | Dietary Supplement |
| 34 | CIMAvax-EGFÂ®-post-COVID19 convalescent with respiratory disorders-adults-Phase II (CORVAXCIM) (RPCEC00000375) | Vaccine |
| 35 | Randomized, double-blind, placebo-controlled clinical trial to evaluate the efficacy and safety of the COMIRNATY vaccine (COVID-19 mRNA vaccine, Pfizer-BioNTech) in people with long COVID (eudract_number:2021-003331-28) | Vaccine |
| 36 | Study to assess the efficacy of montelukast in improving respiratory symptoms in patients with long COVID: E-SPERANZA COVID PROJECT (NCT04695704; eudract_number:2021-000605-24) | Trial registry of a published protocol |
| 37 | Efficacy of Montelukast in Mild-moderate Respiratory Symptoms in Patients with Long-COVID-19 (NCT04695704) | Trial registry of a published protocol |
| 38 | Clinical Trial of Niagen to Examine Recovery in People With Persistent Cognitive and Physical Symptoms After COVID-19 Illness (Long-COVID) (NCT04809974) | Dietary Supplement |

**Reasons for exclusion**: Not on Post-COVID-19 Condition (PCC) = 5, Traditional Chinese Medicine and Ayurvedic medicine =5, Dietary supplement = 8, Study design =8, Stem cell therapy = 4, Phototherapy =1, Plasma/ platelet therapy = 3, on vaccines = 2, trial registry of a published protocol = 2

**F. Risk of bias on included studies**

| **Author and publication year** | **Random sequence generation** | **Allocation concealment** | **Baseline outcome measurements similar** | **Baseline characteristics similar** | **Incomplete outcome data** | **Knowledge of the allocated interventions adequately prevented during the study** | **Protection against contamination** | **Selective outcome reporting** | **Other risks of bias** |
| --- | --- | --- | --- | --- | --- | --- | --- | --- | --- |
| Le Bon et al, 2021 | High | High | Low | Low | Low | Unclear | Low | Low | High  (Volunteer bias) |
| Vaira et al, 2021 | Low | Unclear | Low | Low | Low | Low | Low | Low | Low |
| Abdelalim et al, 2021 | Unclear | Unclear | Low | Low | Low | Unclear | Low | Low | Low |
| Jadhav et al, 2020 | Unclear | High | Unclear | High | Low | Unclear | Low | Low | Low |
| Botek et al, 2022 | Low | Unclear | Low | Low | Low | Unclear | Low | Low | Low |

**G. Comparison of interventions with steroids versus control**

Population: Post-COVID-19 condition

Intervention: Steroids (any) with our without other intervention components (see Forest plot)

Comparator: No treatment or olfactory training

Outcome: Olfactory function at last measured time point


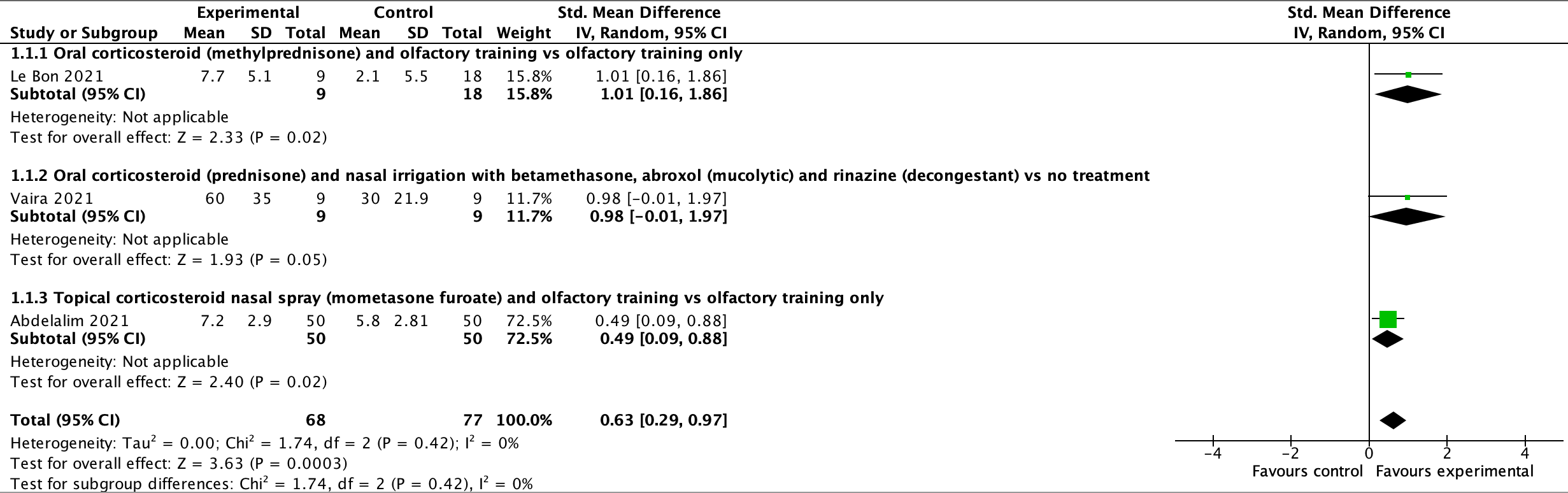


**H**. **Table 3F:** Summary of findings - Effect of corticosteroids for the treatment of people with post-COVID-19 condition

Population: Post-COVID-19 condition

Intervention: Steroids (any) with or without other intervention components

Comparator: No treatment or olfactory training

| **Outcome**  Timeframe | **Study results and measurements** | **Absolute effect estimates** | | **Certainty of the Evidence**  (Quality of evidence) | **Plain language summary** |
| --- | --- | --- | --- | --- | --- |
|  |  | No treatment or olfactory training | Steroids (any) with or without other intervention components |  |  |
| Olfactory function  [Nervous system functioning, symptoms, and condition] | Measured by:  Change in threshold-discrimination-identification (TDI) score (scale: 1 to 48, ≥30.75 (normosmic)  <30.75 (dysosmic), higher better)*  Follow-up: 3-10 weeks  Based on data from 153 participants in 3 studies  Minimal important difference of TDI: 5.5 | 2.1^1^  (mean) | The mean olfactory function score in the intervention groups was **2.7 higher** (1.2 higher to 4.1 higher)^2^ | **Low**  Due to very serious risk of bias^3^ | Steroids (any) with or without other corticosteroids may improve olfactory function |

^1^Mean taken from control group scores of Le Bon (2021), which used TDI scale

^2^Scores calculated based on an SMD of 0.63 higher (CI 95% 0.29 higher to 0.97 higher) and rescaled to TDI 1-48 scale using SD from a larger representative observational study (Oleszkiewicz et al 2019).

**^1^Risk of Bias**: **Very serious in 1 study and serious in 2 studies**. Inadequate sequence generation/ generation of comparable groups, resulting in potential for selection bias, Inadequate concealment of allocation during randomization process, resulting in potential for selection bias, Inadequate/lack of blinding of outcome assessors, resulting in potential for detection bias.
